# Protection of beta-blocker and 5-hydroxytryptamine 1A receptor agonist on myocardial ischemia injury in rats by regulating the NLRP3/Caspase-1/GSDMD pathway

**DOI:** 10.1371/journal.pone.0360695

**Published:** 2026-10-14

**Authors:** Haiyang Chen, Yanwei Li, Meiyan Liu, Yunpeng Chi, Lijun Zhang

**Affiliations:** Department of Psycho-cardiology, Beijing Anzhen Hospital, Capital Medical University, Beijing, China; Versiti Blood Research Institute, UNITED STATES OF AMERICA

## Abstract

**Objective:**

To explore the effects and related mechanisms of a β-blocker combined with a 5-hydroxytryptamine 1A receptor (5-HT1AR) agonist on MI injuries in rats.

**Methods:**

TTC/Evans blue, H&E, Masson, and TUNEL staining were used to assess infarction area, pathological damage, fibrosis, and apoptosis in myocardial tissue. The ELISA kits were used to detect IL-1β and IL-18 levels. The immunohistochemical (IHC) staining was used in conjunction with qRT-PCR and Western blot to determine IL-1β, IL-18, NLRP3, pro Caspase-1, cleaved Caspase-1, GSDMD-FL, and GSDMD-N expression levels.

**Results:**

In myocardial tissue, the β-blocker combined with the 5-HT1AR agonist effectively reduced infarction size, mitigated pathological injury, alleviated fibrosis, and prevented apoptosis. Meanwhile, the β-blocker combined with the 5-HT1AR agonist downregulated IL-1β and IL-18 levels in serum and myocardial tissue. Notably, although it barely altered the expression of the pyroptosis precursor proteins pro Caspase‑1 and GSDMD‑FL, the combined therapy strongly suppressed the mRNA and protein levels of NLRP3, cleaved Caspase‑1, and GSDMD‑N, and reduced the ratios of pro Caspase‑1/cleaved Caspase‑1 and GSDMD-FL/GSDMD-N in myocardial tissue.

**Conclusion:**

β-blocker combined with the 5-HT1AR agonist relieves MI injuries by suppressing the NLRP3/Caspase-1/GSDMD pathway.

## Introduction

Cardiovascular disease, one of the disorders with the highest morbidity and mortality worldwide, has seriously threatened the health of human beings, among which acute myocardial infarction (AMI) is the most notorious [1]. Several pathological changes in myocardial tissues and cells caused by coronary artery occlusion may occur when AMI appears, mainly including dysfunction of myocardial metabolism and inflammatory necrosis [2,3]. Although the application of percutaneous coronary intervention (PCI) significantly reduces the mortality rate of AMI patients, the dual mental stress caused by PCI surgery and long-term critical myocardiopathy generally leads to negative emotions in patients, such as anxiety and depression [4,5]. A large number of studies have demonstrated that emotional disorders, such as anxiety and depression, significantly influence the postoperative recovery and prognosis of patients, which induces the occurrence of multiple cardiovascular adverse events after PCI surgery as well [6,7]. Moreover, some studies also suggested that reducing anxiety, depression, and some other emotions in AMI patients contributed to improving their prognosis and reducing the incidence of stress-related cardiac events [8,9]. Therefore, it is imperative to emphasize the mitigation of mental stress (including anxiety and depression) in patients when consolidating the treatment of cardiovascular diseases.

The inflammatory cascade response is one of the most critical pathological mechanisms during the occurrence and development of AMI [10,11]. Previous studies have shown that inflammasomes, complexes of various proteins and important components of the innate immune system, regulate the inflammatory response by recruiting relevant inflammatory cells during AMI progression, ventricular remodeling, and heart failure [12]. Among the diverse inflammasomes, the NOD-like receptor thermal protein domain-associated protein 3 (NLRP3) inflammasome, composed of NLRP3, apoptosis-related speck-like protein (ASC), and Caspase-1, was investigated in the current study [13]. NLRP3 has three structural domains: leucine-rich repeats (LRRs), the nucleotide-binding and oligomerization domain (NACHT), and the N-terminal pyrin domain, among which NACHT combines with LRRs to inhibit NLRP3 inflammasomes under normal physiological conditions [14]. Under pathological conditions, NLRP3 inflammasomes are overexpressed and activated by nuclear factor kappa-B (NF-κB) during AMI, suggesting that NLRP3 inflammasomes are a potential target for intervention in AMI [15].

Although the cardioprotective effects of bevantolol (BVT), a typical beta-blocker (β-blocker), have been affirmed, its therapeutic mechanisms in AMI remain unclear [16]. Moreover, increasing studies have demonstrated that the management of anxiety and depression contributes to the prognosis of AMI. For example, benzodiazepines improved myocardial metabolite risk factors, such as blood pressure, lipid profile, and glycemic control [17]. Therefore, in the present study, we investigated the effects and mechanisms of combining BVT with tandospirone (TDS, an anti-anxiety agent that targets the 5-hydroxytryptamine 1A receptor) in mitigating MI injury.

## Materials and methods

### Drugs and reagents

BVT and TDS were purchased from Sichuan Credit Pharmaceutical Co., Ltd. (Sichuan, China). Evans blue solution (Cat., E8010) was provided by Solarbio Science & Technology Company (Beijing, China). Hematoxylin & eosin (H&E) staining kit (Cat., G1005), terminal deoxynucleotidyl transferase-mediated dUTP-biotin nick end labeling (TUNEL) assay kit (Cat., G1501), Masson staining kit (Cat., G1006), and TTC dyeing solution (Cat., G1017) were purchased from Servicebio Technology (Wuhan, China). The rats’ enzyme-linked immunosorbent assay (ELISA) kits of creatine kinase-MB (CK-MB) isoenzyme (Cat., MB-6930A), cardiac troponin I (cTn I, Cat., MB-1967A), interleukin-1β (IL-1β, Cat., MB-1588A), and interleukin-18 (IL-18, Cat., MB-1735A) were bought from Meibiao Biology (Nanjing, China). The NovoScript® Plus All-in-one 1st Strand cDNA Synthesis SuperMix (Cat., E047) and NovoStart®Universal Fast SYBR qPCR SuperMix (Cat., E401) were bought from Novoprotein (Shanghai, China). The primary antibodies used for immunohistochemical (IHC) staining and Western blot in the present study are as follows: anti-NLRP3, anti-pro Caspase-1, anti-cleaved Caspase-1, anti-gasdermin-D N-terminal domain (GSDMD-N), anti-GSDMD-full-length (GSDMD-FL), anti-IL-1β, and anti-IL-18. The IL-1β antibody (Cat., AF5103), IL-18 antibody (Cat., DF6252), NLRP3 antibody (Cat., DF15549), cleaved Caspase-1 antibody (Cat., AF4005), and GSDMD-N antibody (Cat., DF13758) were obtained from Affinity (Ohio, USA). The pro Caspase-1 (Cat., ab179515) and GSDMD-FL (Cat., 20770–1-AP) antibodies were bought from Abcam (Cambridge, UK) and Proteintech (Wuhan, China), respectively.

### Rats and animal experimental protocol

Male Sprague-Dawley rats that were provided by Beijing Vital River Laboratory Animal Technology Co., Ltd. (certificate No. SCXK (Jing) 2016–0006) were adaptively cared for in line with the animal care guidelines formulated by the Institute for Laboratory Animal Research of Capital Medical University affiliated with Beijing Anzhen Hospital. During the adaptation period, all rats lived under a 24-hour light-dark cycle at 24°C and about 55% humidity, with free access to food and water. All experimental operations were approved by the Animal Subjects Review Board of the Beijing Institute of Heart Lung and Blood Vessel Diseases (license number: SYXK (Jing) 2021−0042). All rats were randomly divided into three groups (n = 9): Sham, myocardial infarction (MI), and BVT + TDS. Rats in the Sham group underwent sham surgery and received physiological saline (5 mL/kg, i.g.) for one week; namely, the thorax was sutured again after a 6−0 nylon thread was crossed below the left anterior descending (LAD) without ligation. In the other two groups, the 6−0 nylon thread was crossed below the rats’ LAD with ligation before the thorax was stitched again. During and after surgery, the constant-temperature heating pads were used to maintain the body temperature of anesthetized rats. Meanwhile, the postoperative wound care was performed every 24 hours to prevent infection. The difference between the MI group and the BVT + TDS group was that the combination of BVT (10.5 mg/kg, i.g.) and TDS (3.15 mg/kg, i.g.) was administered to rats in the BVT + TDS group for one week, while physiological saline (5 mL/kg, i.g.) was administered to rats in the MI group for one week. The dosages of BVT and TDS were converted from clinical human doses by normalizing to body surface area using the *K*_m_ factor method [18]. The conversion formula was: *D*_rat_ (mg/kg) = *D*_human_ (mg/kg) × (*K*_m, human_/*K*_m, rat_), where *K*_m, human_ = 37 (60 kg adult) and *K*_m, rat_ = 6 [19]. The conversion factor from human dose to rat dose was 6.2. BVT and TDS were dissolved in physiological saline to prepare the suspension of BVT (2.1 mg/mL) and TDS (0.63 mg/mL) before administration. After the last administration, all rats were euthanized with intraperitoneal injection of 2% pentobarbital sodium (60 mg/kg) to achieve deep unconsciousness and eliminate pain perception. The Animal Ethical and Welfare Committee of Capital Medical University affiliated with Beijing Anzhen Hospital approved the experiment under ethical code AZ2023LA007. In each group of nine rats, different numbers of biological replicates were used for subsequent detection experiments.

### TTC/Evans blue staining

In the present study, TTC/Evans blue staining was used to determine myocardial infarct size, following the previous study [20]. Briefly, the rats’ hearts were harvested and stored at −80°C after injecting Evans blue staining solution into the ascending aorta. The frozen hearts were further cut into five 2 mm-thick slices and immersed in TTC staining solution at 37°C for 15 min. The area stained by Evans blue represents the non-ischemic area, and the area stained by the TTC represents the non-infarcted area. The myocardial infarction size was determined using ImageJ (version 1.52a) by calculating the proportion of the infarcted area (unstained TTC) to the ischemic area (unstained part of Evans blue).

### H&E staining

After finishing the one-week combined administration of BVT and TDS, all rats’ hearts were harvested and immersed in 10% paraformaldehyde. The rats’ hearts were then embedded in paraffin wax and cut into 2 μm-thick sections. After dewaxing, sections were stained with hematoxylin and eosin for 5 min at room temperature. All stained sections were scanned digitally to visualize myocardial tissue injury.

### Masson staining

After dewaxing the 2 μm-thick sections of rat hearts, all sections were immersed in Masson A solution overnight. Then, an equal mixture of Masson B and Masson C solutions was used to stain the sections for 1 min, followed by sequential staining with Masson D for 6 min, Masson E for 1 min, and Masson F for 5 s. All stained sections were subjected to digital slice scanning to assess the degree of myocardial fibrosis. Using ImageJ software (version 1.52a), we measured the positively stained collagen area and the total tissue area. The collagen volume fraction (CVF) of myocardial tissue was determined by calculating the proportion of the positively stained collagen area to the total tissue area.

### TUNEL staining

After the 2 μm-thick sections of rats’ hearts were dewaxed, they were first treated with the proteinase K working solution. Then, a 3% hydrogen peroxide solution was used to block the endogenous peroxidase in the sections in the dark for 20 min. The sections were successively incubated with the TUNEL reaction solution and the Streptavidin-HRP reaction solution at 37℃ for 1 h and 30 min, respectively, after the buffer was incubated with the sections for 10 min at room temperature. The DAB solution was used to color the sections, then hematoxylin was finally employed to counterstain the nuclei for 1 min. All stained sections were digitally scanned to assess myocardial apoptosis.

### IHC staining

After the 2 μm-thick sections of rat hearts were dewaxed, antigens were first repaired with sodium citrate solution for 15 min, then 3% hydrogen peroxide and 3% bovine serum albumin were sequentially used to inhibit endogenous peroxidase and remove non-specific adsorption. All sections were immersed in the primary antibodies (anti-NLRP3, 1:300; anti-cleaved Caspase-1, 1:100; anti-GSDMD-N, 1:300) at 4°C before incubation with secondary antibodies (1: 5000) for 50 min at room temperature. After nuclei were counterstained with hematoxylin, all sections were visualized by digital slice scanning to assess NLRP3, cleaved Caspase-1, and GSDMD-N expression in myocardial tissue.

### ELISA assay

After collecting blood from the abdominal aorta of rats and gathering myocardial tissue, the serum was separated at 3000 rpm at 4°C and stored at −80°C until use. The levels of CK-MB, cTn I, IL-1β, and IL-18 in serum, and the levels of IL-1β and IL-18 in the myocardial tissue, were determined using the corresponding ELISA kits according to the manufacturers’ procedures.

### Quantitative reverse transcription-polymerase chain reaction (qRT-PCR)

After extracting total RNA from myocardial tissues using TRIzol reagent, we assessed its concentration and purity and reverse-transcribed it into cDNA using a NovoScript® Plus All-in-one 1st Strand cDNA Synthesis SuperMix. The amplification was accomplished on a fluorescence quantitative PCR instrument (FQD-96A, Bioer, Hangzhou, China) using a NovoStart®Universal Fast SYBR qPCR SuperMix. Sagon (Shanghai, China) designed and synthesized primer sequences for the target genes NLRP3, Caspase-1, GSDMD, IL-18, and IL-1β, listed in Table 1. GAPDH served as the internal standard. The relative mRNA expression levels of each target gene were calculated using the 2^−ΔΔCt^ approach.

**Table 1:** The sequence of primers for qRT-PCR.

mRNA	Forward	Reverse
IL-18	TGAAATAGCAGCTTTCGACAGT	AGATTTGAAGCTGGATGCTCT
IL-1β	TCAGCTCTTCTACCAGCAAAC	ACTTCCAACTGAGAGGCTGT
NLRP3	CGGACTGACCCATCAATGCT	GCAGCTGACCAACCAGAGTT
Caspase-1	GGGCAAAGAGGAAGCAATTTATC	GCCAGGTAGCAGTCTTCATTAC
GSDMD	GGATGCCTGCTTGTTGAGTTG	GACGGCATGATCCTCTTAGG
GAPDH	ACCCTCAACAGGGATGCTTAC	CGATACGGCCAAATCCGTTCA

### Western blot

The harvested myocardial tissue was first transferred into an ice bath with an ultrasound bath for complete lysis after being supplemented with the RIPA lysis solution. After applying the BCA kit to determine the protein concentration, the protein was denatured in boiling water for 15 min. Next, the denatured protein was loaded into a sodium dodecyl sulfate-polyacrylamide gel for electrophoresis to separate proteins by molecular weight. The isolated proteins were transferred from the gel to a polyvinylidene fluoride (PVDF) membrane. The PVDF membrane was immersed in skimmed milk for 30 min to remove non-specific protein adsorption. Then, the PVDF membrane was successively incubated with the primary antibodies (anti-IL-18, 1:1000; anti-IL-1β, 1:1000; anti-NLRP3, 1:1000; anti-pro Caspase-1, 1: 1000; anti-cleaved Caspase-1, 1:1000; anti-GSDMD-FL (1: 2000); anti-GSDMD-N, 1:1000; anti-GAPDH, 1:3000) at 4°C overnight and with the secondary antibodies (1: 5000) for 30 min at room temperature. After incubating the PVDF membrane with the ECL luminescent solution for 1 min, the blots were imaged using a chemiluminescence imaging system, and the gray values were quantified with AIWBwell^TM^ analysis software to determine target protein expression levels in myocardial tissue. We used distinct domain‑specific primary antibodies to detect pro Caspase‑1 (pro‑domain epitope), cleaved Caspase‑1 (catalytic‑subunit epitope), GSDMD‑FL (C‑terminal‑domain epitope), and GSDMD‑N (N‑terminal‑domain epitope) separately, because a single antibody cannot reliably distinguish full‑length precursors from their proteolytically cleaved active fragments in rat myocardial tissue.

### Statistical analysis

All data from independent triplicate experiments were presented as mean ± standard deviation (SD). In this study, nine rats were enrolled per experimental group (n = 9 biological replicates). For histological staining‑based quantitative analyses, including TTC/Evans blue, H&E, Masson, TUNEL, and IHC staining, three biological replicates were selected for statistical quantification (n = 3). For serum ELISA detection of CK‑MB, cTn I, IL‑1β, and IL‑18, six biological replicates were used (n = 6). Western blot assays were performed with three biological replicates (n = 3). With the help of GraphPad (v. 8.0.2), the difference between groups was evaluated by adopting a one-way analysis of variance, and the experimental data were transferred into the form of figures. *P* < 0.05 indicates statistically significant differences.

## Results

### BVT combined with TDS reduces the size of the myocardial infarction

After analyzing the unstained areas of Evans blue and TTC, we calculated the myocardial infarction size in each group. As presented in Fig 1A and 1B, myocardial infarction size in the MI group was significantly increased compared with the Sham group. Meanwhile, compared with the MI group, the myocardial infarction size in the BVT + TDS group was prominently reduced. These results suggest that combined administration of BVT and TDS significantly reduced myocardial infarction size in rats.

**Fig 1:**
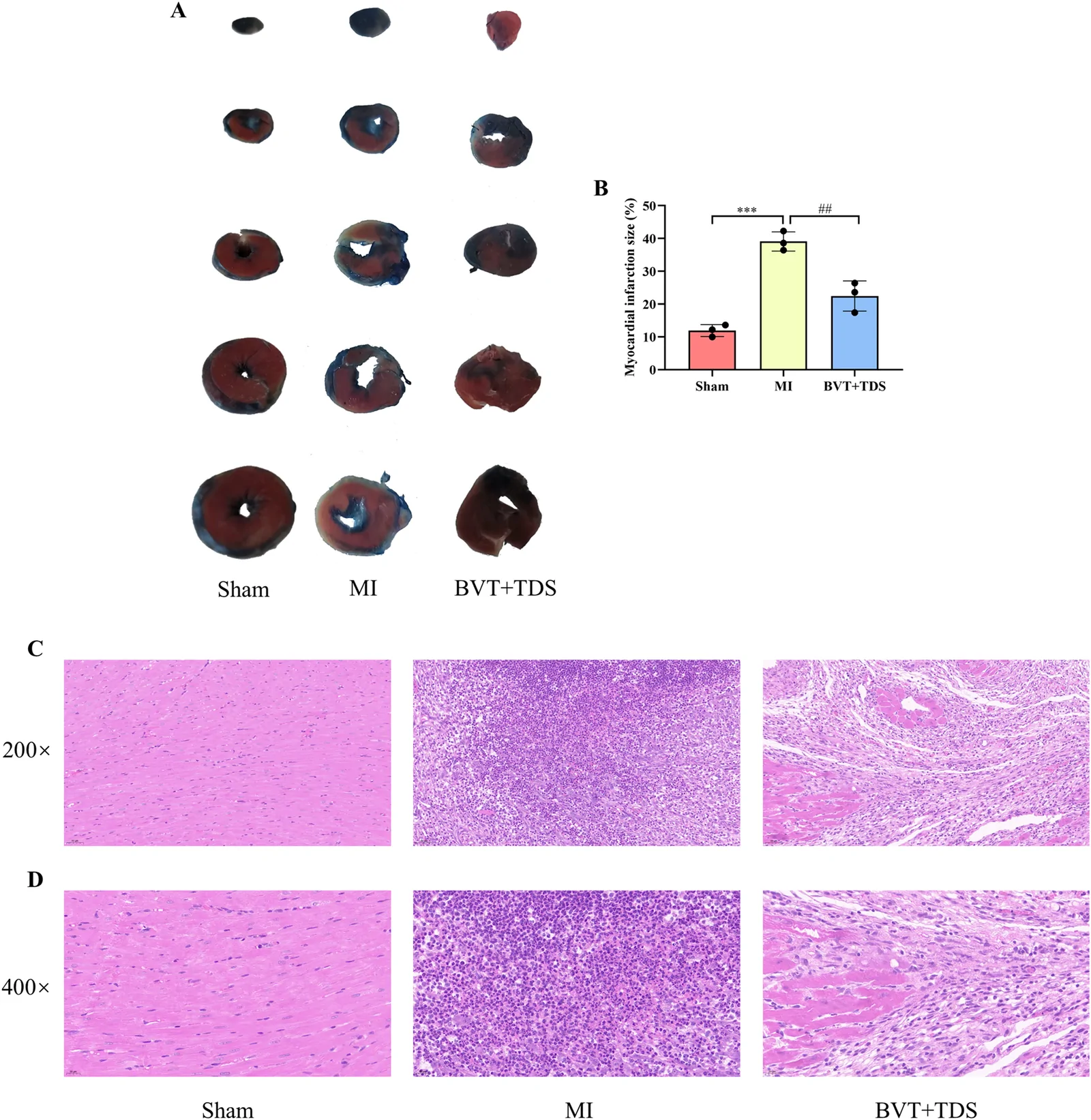
Effect of BVT combined with TDS on myocardial infarction size and myocardial pathological injury. (**A**) Diagrams of TTC/Evans blue double staining of rats’ myocardial tissue in each group. (**B**) Quantitative analysis of myocardial infarction size in rats. (**C**) H&E-stained rat myocardial tissue in each group was observed at 200 × magnification (scale bar: 50 μm). (**D**) H&E-stained rat myocardial tissue from each group was observed at 400 × (scale bar: 20 μm). All experimental data were expressed as mean ± SD (*n* = 3, biological replicates). ^***^p < 0.001 vs. Sham; ^##^*p* < 0.01 vs. MI. Note: No *p*hysical ruler was embedded during *p*hotograph acquisition for these gross‑view myocardial slices. We quantified infarct size using a calibrated scale function built into ImageJ rather than manual ruler measurement. Size variation among myocardial tissue samples mainly arises from biological differences rather than tissue preparation or image acquisition.

### BVT combined with TDS alleviates myocardial pathological damage

H&E staining was further performed to investigate the effects of combined BVT and TDS treatment on rat myocardial tissue. As shown in Fig 1C (at 200 × magnification), myocardial tissue was directionally and orderly arranged in the Sham group. In the MI group, the myocardial tissue was disorganized, with abundant inflammatory cells infiltrating around cardiomyocytes, numerous collagenous fibers, and an obvious cicatrix. Compared with the MI group, the BVT + TDS group showed markedly reduced inflammatory infiltration, collagenous fibers, and cicatrix. As shown in Fig 1D (at 400 × magnification), cardiomyocytes in the Sham group had a long shuttle shape. In the MI group, the endochylema of cardiomyocytes dissolved, the cardiomyocytes showed karyopyknosis, and extensive neutrophils, macrophages, and monocytes were distributed around the cardiomyocytes. Compared with the MI group, the histopathological injuries described above were significantly mitigated. These results demonstrate that combined BVT and TDS treatment effectively alleviates pathological damage to myocardial tissue.

### BVT combined with TDS mitigates myocardial fibrosis

Using Masson staining, myocardial collagen fibers were stained blue, and myocardial tissue was stained red. As presented in Fig 2A and 2B, compared with the Sham group, there were massively hyperplastic collagenous fibers in the myocardial tissue, and the CVF value was dramatically upregulated in the MI group. The area of the blue-stained part of myocardial tissue and the CVF value in the BVT + TDS group were prominently reduced compared with those in the MI group. The above results indicated that BVT combined with TDS substantially prevented myocardial fibrosis.

**Fig 2:**
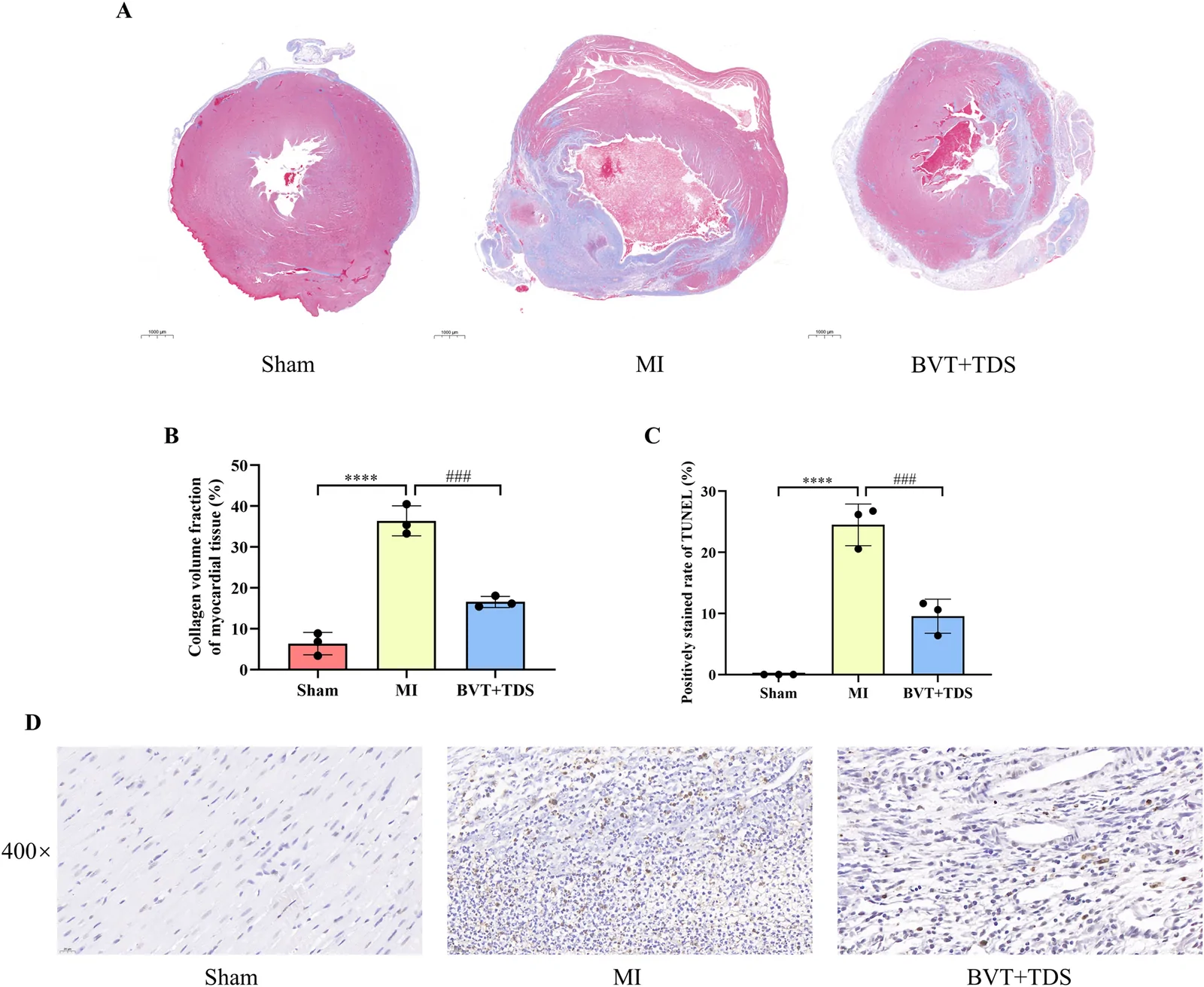
Effect of BVT combined with TDS on myocardial fibrosis and myocardial apoptosis. (**A**) Masson staining diagrams of rats’ myocardial tissue in each group at 10 × magnification (scale bar: 1000 μm). (**B**) Quantitative analysis of the CVF value in rats’ myocardial tissue. All experimental data were expressed as mean ± SD (*n* = 3, biological replicates). (**C**) Quantitative analysis of the TUNEL-positive staining rate in rat myocardial tissue. All experimental data were expressed as mean ± SD (*n* = 3, biological replicates). (**D**) TUNEL staining diagrams of rats’ myocardial tissue in each group at 400 × magnification (scale bar: 20 μm). ^****^p < 0.0001 vs. Sham; ^###^*p* < 0.001 vs. MI.

### BVT combined with TDS suppressed myocardial apoptosis

TUNEL staining was subsequently performed to investigate the effect of BVT combined with TDS on apoptosis in rat myocardial tissue. As shown in Fig 2D, brown-stained cells represented apoptotic cells, while blue-stained cells represented non-apoptotic cells. As shown in Fig 2C, the TUNEL‑positive cell rate in myocardial tissue from the MI group was demonstrably higher than in the Sham group. Compared with the MI group, the number of apoptotic cells in the BVT + TDS group significantly declined at the same magnification. The above results indicated that BVT combined with TDS effectively inhibited myocardial apoptosis.

### BVT combined with TDS decreased the levels of myocardial injury markers and inflammatory cytokines

We subsequently investigated the effect of BVT combined with TDS on the levels of myocardial injury markers and inflammatory cytokines in serum and myocardial tissue because the inflammatory response is a primary driver of AMI [21]. As shown in Fig 3A-3F, the trends in the levels of myocardial injury markers and inflammatory cytokines in serum and myocardial tissue were similar. Compared with the Sham group, the MI group showed dramatically higher levels of myocardial injury markers and inflammatory cytokines in serum and myocardial tissue. However, in the BVT + TDS group, the levels of myocardial injury markers and inflammatory cytokines in serum and myocardial tissue in the BVT + TDS group were markedly lower than in the MI group. Moreover, as illustrated in Fig 5A, 5C and 5D, the mRNA and protein expression levels of IL-18 and IL-1β were dramatically upregulated in the MI group compared with the Sham group. Compared with the MI group, the mRNA and protein expression levels of IL-18 and IL-1β in the BVT + TDS group were prominently down-regulated. These results indicated that BVT combined with TDS significantly down-regulated myocardial injury markers and inflammatory cytokines.

**Fig 3:**
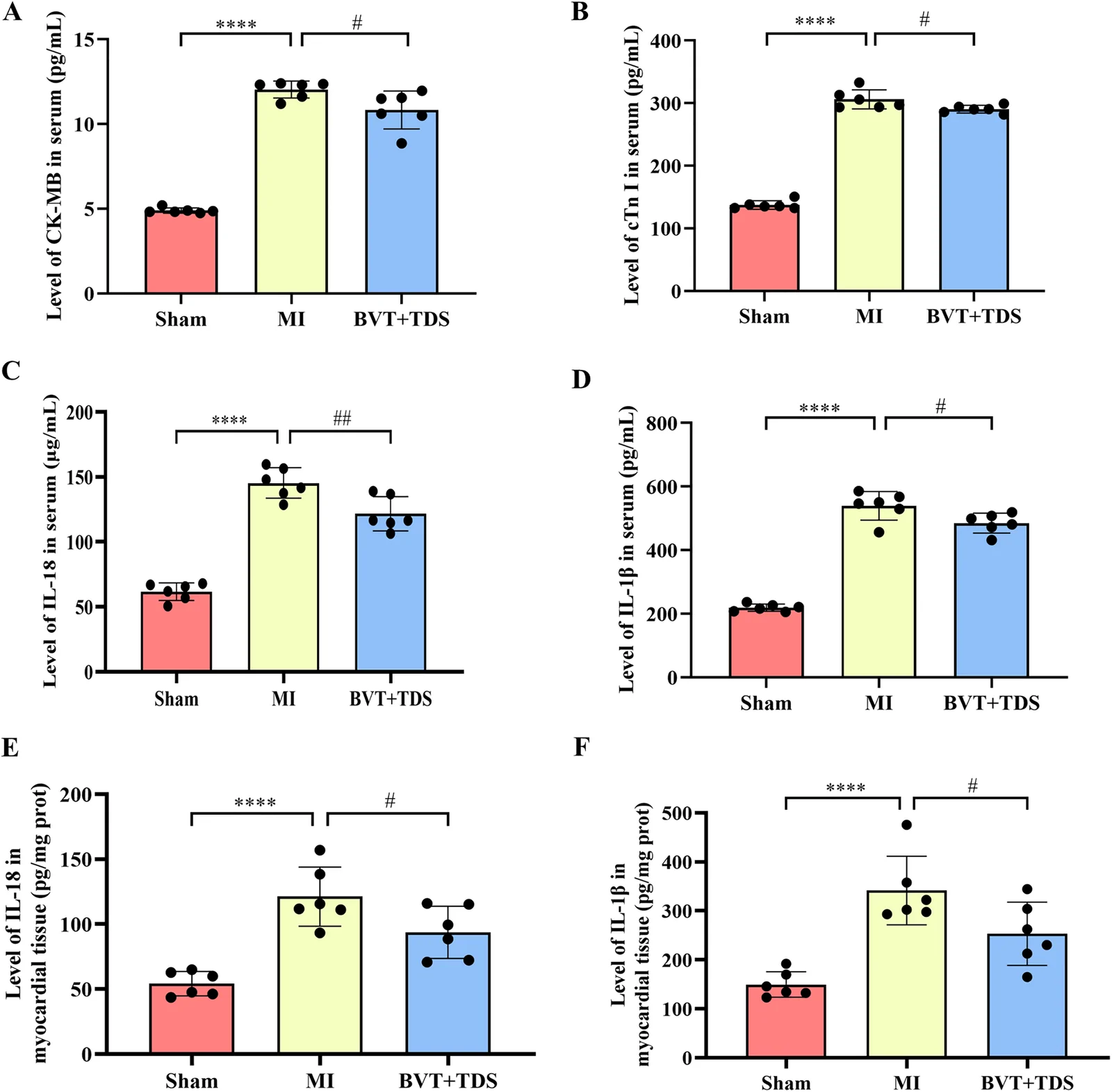
Effect of BVT combined with TDS on myocardial injury markers and inflammatory cytokines in serum and myocardial tissue. The levels of CK-MB (**A**) and cTnI (**B**) in serum. The levels of IL-18 in serum (**C**) and myocardial tissue (**E**). The levels of IL-1β in serum (**D**) and myocardial tissue (**F**). All experimental data were expressed as mean ± SD (*n* = 6, biological replicates). ^****^p < 0.0001 vs. Sham; ^##/#^*p* < 0.01/0.05 vs. MI.

### BVT combined with TDS restrained the NLRP3/Caspase-1/GSDMD pathway

To investigate whether combined BVT and TDS reduce myocardial injury by regulating the NLRP3/Caspase-1/GSDMD pathway, we further used IHC staining and Western blot in this study. As presented in Fig 4A, 4C, and 4E (at 400 × magnification), the brown-stained areas represent positive expression of the related proteins (including NLRP3, cleaved Caspase-1, and GSDMD-N). Fig 4B, 4D, and 4F show the area analysis of the positively stained regions for the three proteins in each group. Compared with the Sham group, the positively stained area of the three proteins was significantly enhanced in the MI group. BVT combined with TDS administration effectively reduced the positively stained area of the three proteins in myocardial tissue. In addition, as illustrated in Fig 5B, 5G, and 5H, the trends in the mRNA and protein expression levels of NLRP3, cleaved Caspase-1, and GSDMD-N matched the IHC staining results. In short, the mRNA and protein expression levels of NLRP3, cleaved Caspase-1, and GSDMD-N in the MI group were significantly upregulated compared with those in the Sham group. The expression levels of these genes and proteins were dramatically down-regulated in the BVT + TDS group compared with the MI group. In addition, we detected protein expression levels of GSDMD-FL and pro Caspase-1 in myocardial tissue to assess the extent of protein cleavage and activation. As shown in Fig 5E and 5F, compared with the Sham group, GSDMD-FL and pro Caspase-1 protein expression levels were significantly upregulated in the MI group. However, although the protein expression levels of GSDMD-FL and pro Caspase-1 in the BVT + TDS group were lower than those in the MI group, there were no significant differences. Based on the protein expression levels of pro Caspase-1, cleaved Caspase-1, GSDMD-FL, and GSDMD-N in each group, we calculated the proportion of cleaved Caspase-1 to pro Caspase-1 and GSDMD-N to GSDMD-FL. As illustrated in Fig 5I, compared with the Sham group, the MI group showed significantly increased ratios of cleaved Caspase-1/pro Caspase-1 and GSDMD-N/GSDMD-FL. Combined BVT and TDS significantly reduced these ratios, indicating that the combined treatment suppressed caspase-1 activation and subsequent GSDMD cleavage after myocardial infarction. The above results indicated that the mechanism of the combined treatment of BVT and TDS in treating myocardial injuries caused by AMI may be involved in the NLRP3/Caspase-1/GSDMD pathway.

**Fig 4:**
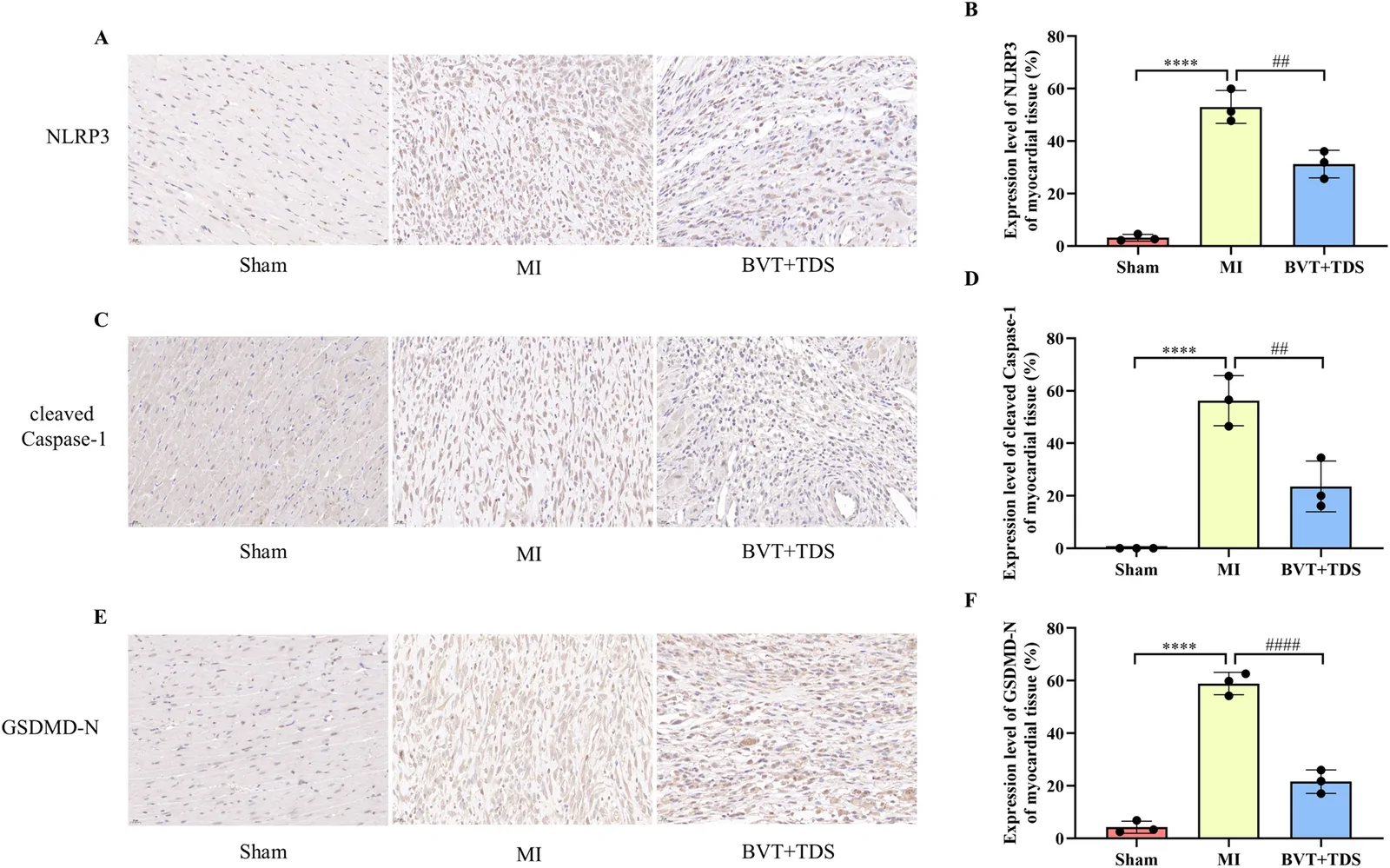
Effect of BVT combined with TDS on the expression of related proteins in the myocardial tissue. (**A**) IHC staining diagrams of NLRP3 in rat myocardial tissue for each group at 400 × magnification (scale bar: 20 μm). (**B**) Quantitative analysis of NLRP3 expression. (**C**) IHC staining diagrams of cleaved Caspase-1 in rats’ myocardial tissue in each group at 400 × (scale bar: 20 μm). (**D**) Quantitative analysis of cleaved Caspase-1 expression. (**E**) IHC staining diagrams of GSDMD-N in rats’ myocardial tissue in each group at 400 × (scale bar: 20 μm). (**F**) Quantitative analysis of GSDMD-N expression. All experimental data were expressed as mean ± SD (*n* = 3, biological replicates). ^****^p < 0.0001 vs. Sham; ^####/##^*p* < 0.0001/0.01 vs. MI.

**Fig 5:**
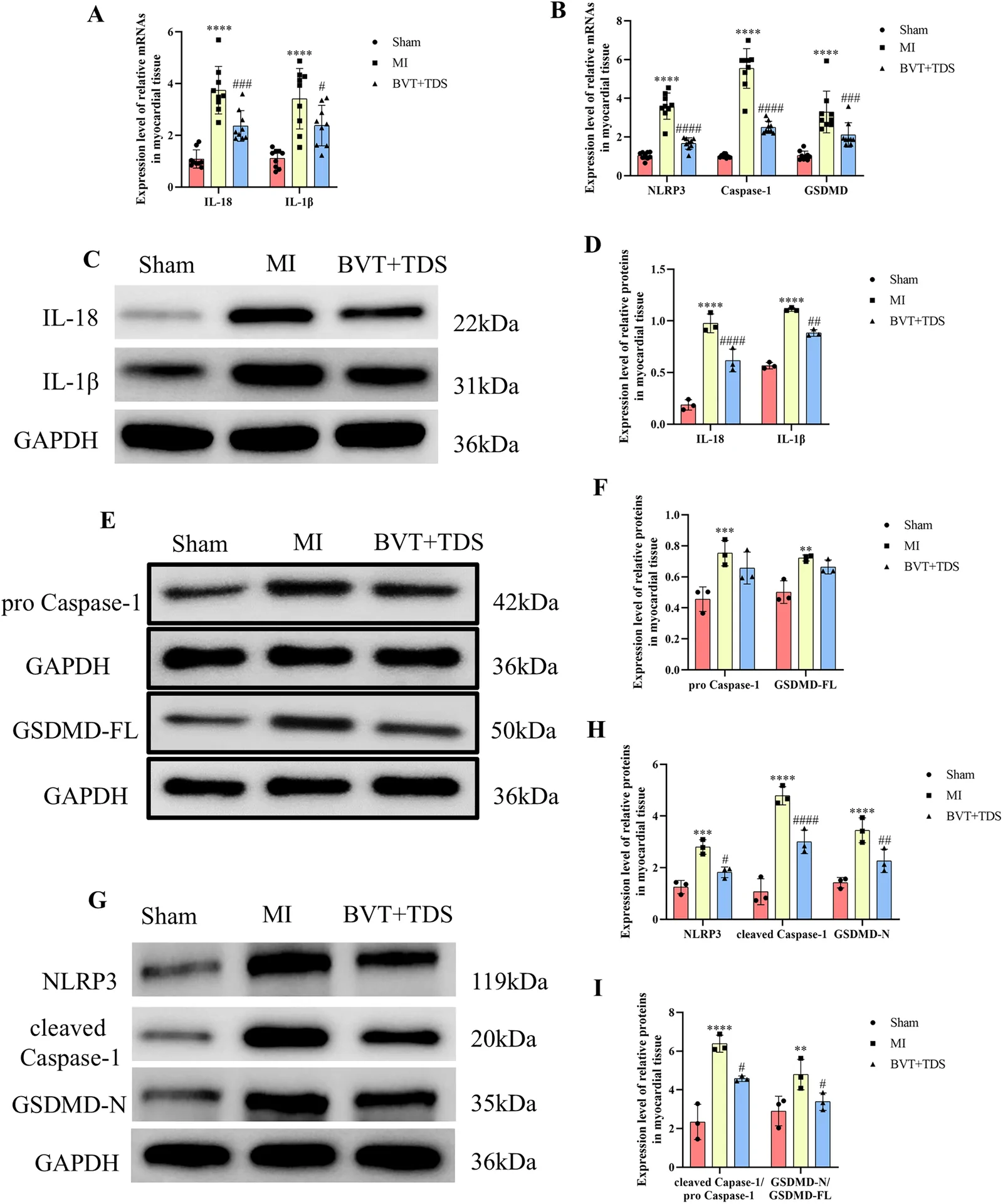
Effect of BVT combined with TDS on the expression level of related genes and proteins in the myocardial tissue. The mRNA expression levels of IL-18 and IL-1β (**A**), as well as NLRP3, Caspase-1, and GSDMD (**B**), in myocardial tissue. (**C**) Protein blot of IL-18, IL-1β, and GAPDH. (**D**) Quantitative analysis of the relative protein expression levels of IL-18 and IL-1β. (**E**) Protein blots of pro Caspase-1, GSDMD-FL, and GAPDH. (**F**) Quantitative analysis of the relative protein expression levels of pro Caspase-1 and GSDMD-FL. (**G**) Protein blots of NLRP3, cleaved Caspase-1, GSDMD-N, and GAPDH. (**H**) Quantitative analysis of the relative protein expression levels of NLRP3, cleaved Caspase-1, and GSDMD-N. (**I**) Ratio of relative protein expression levels of pro Caspase-1/cleaved Caspase-1 and GSDMD-FL/GSDMD-N. All experimental data were expressed as mean ± SD (*n* = 3, biological replicates). ^****/***/**^p < 0.0001/0.001/0.01 vs. Sham; ^####/###/##/#^*p* < 0.0001/0.001/0.01/0.05 vs. MI.

## Discussion

Recently, it was acknowledged that although the mortality rate of AMI has been significantly controlled with the development of interventional and thrombolytic therapy, the occurrence of anxiety and depression after AMI not only diminishes the curative effect of intervention and thrombolysis but also hinders the prognosis of patients and increases the incidence of various serious complications, which cannot be ignored [22,23]. The overstimulated sympathetic nerve by anxiety and depression not only increases the heart rate and elevates blood pressure to enhance myocardial oxygen consumption but also causes the contraction of the coronary artery to reduce myocardial blood supply and exacerbate the rupture of unstable plaques, all of which promote the occurrence and development of AMI [24,25]. Therefore, the timely diagnosis and effective treatment of anxiety and depression play a significant role in improving disease prognosis and reducing the mortality of AMI.

β-blockers have been confirmed to possess cardioprotective effects. The main mechanisms by which β-blockers protect the myocardium include slowing heart rate, retarding myocardial contraction, reducing myocardial oxygen consumption, lowering blood pressure, and extending the diastolic period of the heart [26]. It was also affirmed that β-blockers suppressed the NLRP3 inflammasome and reduced levels of inflammatory cytokines, such as TNF-α and IL-1β [27,28]. Although cardioprotection of β-blockers has been acknowledged, few studies have examined their anti-inflammatory effects and related mechanisms in treating AMI. 5-HT1AR agonists effectively suppress hyperactive 5-HT nerve activity by promoting 5-HT binding to the 5-HT1A receptor on the postsynaptic membrane, exerting anxiolytic effects [29]. In addition, although one previous clinical study showed that 5-HT1AR agonists effectively regulated inflammatory factors in the body, including IL-6 and IL-10, the mechanisms by which 5-HT1AR agonists modulate the inflammatory response remain unknown [30]. Moreover, due to the non-negligible role of accompanying mental disorders (such as anxiety and depression) in the treatment of AMI, it is necessary to combine the commonly used drugs for AMI (such as β-blockers) with anti-anxiety agents or antidepressants for the treatment of AMI in the clinic. Therefore, in this study, we investigated the effects of combining BVT and TDS in alleviating MI injury in rats using multiple approaches, including TTC/Evans blue staining, H&E staining, Masson staining, TUNEL staining, ELISA, and Western blot. The results indicated that the β-blocker combined with the 5-HT1AR agonist effectively reduced the myocardial infarction size, alleviated myocardial pathological injuries, mitigated myocardial fibrosis, prevented myocardial apoptosis, diminished the levels of IL-1β and IL-18 in serum and myocardial tissue, and decreased the mRNA and protein expression levels of IL-1β and IL-18 in myocardial tissue. This shows that β-blocker combined with the 5-HT1AR agonist relieves AMI-induced myocardial injury in rats, likely through their anti-inflammatory effects.

The inflammatory response plays a significant role in the progression of AMI. One previous study has shown that the release of inflammatory cytokines aggravates AMI by affecting atherosclerotic plaque stability [31]. In addition, pyroptosis, a form of cell death closely linked to the body’s innate immune response, is characterized by changes in cell membrane permeability and the extracellular release of inflammatory cytokines [32]. Pyroptosis activation involves three pathways: typical pathways dependent on caspase-1, atypical pathways dependent on caspase-4/5/11, and recently discovered pathways dependent on caspase-8 [33,34]. However, GSDMD is the direct substrate of all downstream caspase families involved in regulating pyroptosis [35]. In recent years, growing evidence confirms that regulating GSDMD-mediated pyroptosis contributes significantly to treating AMI [36,37]. In addition, one previous study found that AMI activates the inflammasome complex composed of NLRP3, ASC, and caspase-1 to trigger GSDMD-mediated pyroptosis of cardiomyocytes [38]. Recent studies have suggested that the immune-inflammatory system is also related to the pathology and physiology of the occurrence and development of anxiety and depression [39,40]. The long-term state of anxiety and depression promotes the release of inflammatory cytokines from the amygdala and hippocampus in the brain to increase inflammatory responses [41]. Furthermore, suppressing the protein expression levels of NLRP3, Caspase-1, and GSDMD mitigates pyroptosis in the prefrontal cortex, which is essential in treating generalized anxiety disorder [42]. Therefore, in the present study, we investigated the novel mechanism by which BVT combined with TDS alleviates MI injury in rats through the NLRP3/Caspase-1/GSDMD pathway. As shown by IHC staining, qRT-PCR, and Western blot results, compared with the Sham group, the positively stained area, mRNA levels, and the grayscale values of protein blots for NLRP3, cleaved Caspase-1, and GSDMD-N in the MI group were all markedly upregulated. The above-mentioned indicators in the BVT + TDS group were significantly downregulated compared with the MI group. Further protein expression analysis of key pyroptotic precursors showed that GSDMD-FL and pro Caspase-1 protein levels were also markedly elevated in the MI group relative to the Sham group. Notably, BVT combined with TDS treatment could not significantly reverse the MI-induced upregulation of GSDMD-FL and pro Caspase-1. However, further quantitative analysis of protein cleavage and activation efficiency revealed that the ratio of cleaved Caspase-1/pro Caspase-1 and GSDMD-N/GSDMD-FL was markedly increased in the MI group, indicating robust activation of myocardial pyroptosis after infarction. Combined BVT and TDS treatment reversed this change and significantly reduced both ratios. These results indicate that combined treatment with a β‑blocker and a 5‑HT1AR agonist barely alters the basal abundance of pro Caspase‑1 and GSDMD‑FL precursor proteins, but markedly restrains their proteolytic cleavage and activation. This effect contributes to the suppression of the NLRP3/Caspase‑1/GSDMD pathway and ultimately ameliorates AMI‑triggered myocardial injury. Our TUNEL assay showed increased DNA-fragmented cell death after MI, which may not be unique to apoptosis. Pyroptosis can also trigger nuclear DNA breakdown and produce positive TUNEL signals, indicating that our in vivo observations reflect the complex cell death output of multiple programmed cell death pathways [43]. Based on our current experimental design, we cannot clearly quantify the relative contribution of apoptosis and NLRP3/Caspase-1/GSDMD-dependent pyroptosis to total cardiomyocyte loss in this MI model without additional pathway-specific blockade or lineage tracing analysis. Our drug intervention simultaneously suppressed molecular markers of pyroptosis and reduced TUNEL-positive cell counts, supporting the possibility of activation of both cell death modes after myocardial infarction and their alleviation through BVT combined with TDS treatment. However, this manuscript focuses on the NLRP3/Caspase-1/GSDMD pathway. Exploring the exact proportion of cell death caused by each pathway will require specialized functional loss experiments targeting apoptosis- and pyroptosis-specific molecules.

So far, we have revealed one mechanism by which combination treatment with a β-blocker and a 5-HT1AR agonist mitigates MI injury in rats. We demonstrated that the β-blocker combined with the 5-HT1AR agonist reduced the myocardial infarction size, alleviated myocardial pathological injuries, mitigated myocardial fibrosis, prevented myocardial apoptosis, diminished the level of IL-1β and IL-18 in serum, and decreased the protein expression level of IL-1β and IL-18 in myocardial tissue by suppressing the NLRP3/Caspase-1/GSDMD pathway. However, the present study still has some limitations. First, we selected only TDS, a 5‑HT1A partial agonist with anxiolytic properties, as the stress‑relieving agent. We did not evaluate other stress‑targeting antagonists or anxiolytic agents in our experimental setup. Therefore, we cannot compare whether alternative stress antagonists exert similar myocardial‑protective and pyroptosis‑inhibitory effects under myocardial‑ischemia conditions, which is another experimental gap to address in future work. Additionally, it is unclear whether the curative effect of combined treatment with a β-blocker and a 5-HT1AR agonist is superior to single-agent β-blocker treatment in AMI. Moreover, it is unclear whether the inhibitory effects on the NLRP3/Caspase-1/GSDMD pathway result from the combined effect of the β-blocker and the 5-HT1AR agonist or from either one alone. To address these issues, we believe subsequent studies should add two independent monotherapy comparison groups to the MI model: the MI + BVT‑alone group and the MI + TDS‑alone group, alongside the existing combination‑therapy group. Secondly, our therapeutic intervention lasted only one week, representing a relatively short treatment window. Therefore, we cannot determine whether the observed myocardial protective phenotypes are transient or can be sustained over longer periods. Furthermore, although our present findings showed that BVT combined with TDS ameliorated myocardial morphological injury (reflected in reduced infarct size, pathological damage, fibrosis, and apoptosis) and myocardial damage (reflected in reduced CK‑MB and cTnI levels), we did not assess cardiac functional parameters in the current work. We lacked long‑term follow‑up measurements, including serial echocardiography to assess left‑ventricular systolic and diastolic function and survival‑rate analysis, which are critical endpoints for judging the durability of cardioprotection and the clinical translational value of this combined regimen. It remains unclear whether BVT + TDS treatment improves long‑term cardiac function and survival outcomes, and future long‑term observational studies with echocardiographic monitoring and survival statistics are warranted. Thirdly, the anxiety-like or depression-like behavior of rats after AMI was not determined in the present study. We consider that behavioral testing of rats and related experimental assays of rats’ brains ought also to be conducted in subsequent studies to investigate the effects of the combination of the β-blocker and the 5-HT1AR agonist on AMI with anxiety or depression. Fourthly, the mental stress model may be supplemented to establish combinational models, which are more similar to the state of clinical patients with AMI and anxiety or depression. Therefore, in the subsequent study, the mental stress model ought to be added to further investigate the effects of the β-blocker and the 5-HT1AR agonist on rats with AMI and anxiety or depression. In addition, although we determined the effects and related mechanisms of the β-blocker-5-HT1AR agonist combination on AMI *in vivo*, the cardioprotection of the β-blocker combined with the 5-HT1AR agonist *in vitro* remains unclear. Therefore, we consider it extremely important to conduct cellular experiments to determine whether the cardioprotective effects of the β-blocker combined with the 5-HT1AR agonist are achieved by inhibiting the NLRP3/Caspase-1/GSDMD pathway *in vitro*. Moreover, although we measured inflammatory cytokines such as IL-18 and IL-1β and the expression levels of NLRP3, cleaved caspase-1, and GSDMD-N, this manuscript does not adequately investigate the causality between the treatment effects of the β-blocker combined with the 5-HT1AR agonist and the inflammatory mechanism. We did not use a well‑established NLRP3 inflammasome inhibitor as a positive control in the current experimental design, which represents an important limitation in confirming the causal role of the NLRP3/Caspase-1/GSDMD pathway. A direct comparison with NLRP3-specific inhibition will enhance the mechanistic explanation and distinguish pathway-specific effects from off-target drug effects. Therefore, we plan to use an NLRP3 inhibitor or activator in subsequent *in vivo* and *in vitro* studies to further confirm the link between the treatment effects of BVT combined with TDS and the inflammatory mechanism. Additionally, because TUNEL staining cannot distinguish apoptosis from pyroptosis, current research cannot quantitatively separate the contributions of apoptosis and pyroptosis to total myocardial cell damage. Future mechanistic experiments should use specific genetic or pharmacological inhibition to analyze the roles of each pathway. Finally, in the present study, we measured total myocardial tissue protein levels of pyroptosis‑related molecules (NLRP3, cleaved Caspase‑1, and GSDMD‑N), we did not perform definitive functional assays to directly confirm pyroptosis. Therefore, we cannot conclude that pyroptosis is definitively inhibited. In the future, we will perform complementary in vitro and in vivo functional experiments to confirm pyroptosis activation, including lactate dehydrogenase release, propidium iodide uptake, and extracellular secretion of mature IL‑1β. In addition, it remains unclear whether cardiomyocytes, inflammatory cells (such as macrophages and neutrophils), or other resident cardiac cells are the major cell types contributing to regulating the NLRP3/Caspase‑1/GSDMD pathway in the MI model. Because we did not perform co‑immunofluorescence staining between NLRP3 and cell‑type‑specific markers (such as the cardiomyocyte marker cTnI, the macrophage marker CD68, and the neutrophil marker Ly6G), we cannot pinpoint the principal cell population driving pyroptosis in infarcted myocardium. Future co-staining experiments will help clarify the cellular origin of pyroptotic signals and further dissect the cell-specific mechanisms underlying the cardioprotective effect of BVT combined with TDS.

Myocardial ischemic injury is closely related to anxiety and depression, which can significantly worsen the prognosis of patients with AMI. Beta blockers are classic drugs used to treat acute myocardial infarction, and 5-HT1AR agonists have anti-anxiety effects. Notably, this work cannot distinguish monodrug effects from synergistic interactions and has not tested other stress‑modulating antagonists. This study indicates that combining a β-blocker with a 5-HT1AR agonist effectively reduces myocardial ischemic injury in rats by inhibiting the NLRP3/Caspase-1/GSDMD pathway and reducing myocardial infarction area, pathological damage, fibrosis, apoptosis, and inflammatory response. These findings provide a new treatment strategy for acute myocardial infarction combined with anxiety and depression in clinical practice, and provide experimental evidence for the combined use of cardiovascular protective drugs and anti-anxiety drugs in clinical practice. However, further animal studies incorporating BVT‑alone and TDS‑alone arms, as well as testing alternative stress‑targeting agents, are required before clinical translational inference can be firmly established. In addition, future work should include a validated NLRP3 inflammasome inhibitor as a positive‑control group to provide more definitive mechanistic evidence linking the cardioprotective effects of this combined regimen to the NLRP3/Caspase‑1/GSDMD pathway and downstream pyroptotic cell‑death events.

## Supporting information

S1 File Raw quantitative data supporting the analyses presented in Fig. 1B, including myocardial infarction size (%). (XLSX)

S2 File Raw quantitative data supporting the analyses presented in Fig. 2, including collagen volume fraction of myocardial tissue (%) and positively staining rate of TUNEL (%). (XLSX)

S3 File Raw quantitative data supporting the analyses presented in Fig. 3, including levels of CK-MB, cTn I, IL-18, and IL-1β in serum and IL-18 and IL-1β in myocardial tissue. (XLSX)

S4 File Raw quantitative data supporting the analyses presented in Fig. 4, including expression levels of NLRP3, cleaved Caspase-1, and GSDMD-N in myocardial tissue (%). (XLSX)

S5 File Raw quantitative data supporting the analyses presented in Fig. 5A and B, including relative mRNA expression levels of IL-18, IL-1β, NLRP3, Caspase-1, and GSDMD in myocardial tissue. (XLSX)

S6 File Raw quantitative data supporting the analyses presented in Fig. 5D, F, H, and I, including relative protein expression levels of IL-18, IL-1β, NLRP3, pro Caspase-1, cleaved Caspase-1, GSDMD-FL, GSDMD-N, cleaved Caspase-1/pro Caspase-1, and GSDMD-N/GSDMD-FL in myocardial tissue. (XLSX)

S7 File Raw data gel image. Uncropped and unedited original gel images corresponding to the Western blot experiments presented in **Fig. 5**.
(PDF)

## Abbreviations

AMI: acute myocardial infarction
AIS: acute ischemic stroke
ASC: apoptosis-related spot protein
β-blocker: beta-blocker
BVT: bevantolol
CK-MB: creatine kinase-MB
cTn I: cardiac troponin I
CVF: collagen volume fraction
ELISA: enzyme-linked immunosorbent assay
GSDMD-N: gasdermin-D-N-terminal domain
H&E: hematoxylin & eosin
5-HT1AR: 5-hydroxytryptamine 1A receptor
IHC: immunohistochemical
IL-1β: interleukin-1β
IL-18: interleukin-18
LAD: left anterior descending
LRRs: leucine-rich-repeats
NACHT: nucleotide-binding and oligomerization domain
NF-κB: nuclear factor kappa-B
NLRP3: NOD-like receptor thermal protein domain associated protein 3
PCI: percutaneous coronary intervention
PSD: post-stroke depression
PVDF: polyvinylidene fluoride
TDS: tandospirone
TUNEL: terminal deoxynucleotidyl transferase-mediated dUTP-biotin nick end labeling

## References

[1] Price S, Katz J, Kaufmann CC, Huber K. The year in cardiovascular medicine 2021: Acute cardiovascular care and ischaemic heart disease. Eur Heart J. 2022;43(8):800–6. doi: 10.1093/eurheartj/ehab908 34977923 PMC9383154

[2] Zuurbier CJ, Bertrand L, Beauloye CR, Andreadou I, Ruiz-Meana M, Jespersen NR, et al. Cardiac metabolism as a driver and therapeutic target of myocardial infarction. J Cell Mol Med. 2020;24(11):5937–54. doi: 10.1111/jcmm.15180 32384583 PMC7294140

[3] Peet C, Ivetic A, Bromage DI, Shah AM. Cardiac monocytes and macrophages after myocardial infarction. Cardiovasc Res. 2020;116(6):1101–12. doi: 10.1093/cvr/cvz336 31841135 PMC7177720

[4] Zheng Y, Hu Y, Han Z, Yan F, Zhang S, Yang Z, et al. Lomitapide ameliorates middle cerebral artery occlusion-induced cerebral ischemia/reperfusion injury by promoting neuronal autophagy and inhibiting microglial migration. CNS Neurosci Ther. 2022;28(12):2183–94. doi: 10.1111/cns.13961 36052650 PMC9627359

[5] Yang Y, Xia L, Li Y, Duan T, Lv X, Wang Y. Effects of meditation and breathing therapy on anxiety improvement before and after percutaneous coronary intervention in patients with coronary artery disease: A prospective cohort study. Heart and Mind. 2024;8(3):239–47. doi: 10.4103/hm.hm-d-24-00027

[6] Kala P, Hudakova N, Jurajda M, Kasparek T, Ustohal L, Parenica J, et al. Depression and anxiety after acute myocardial infarction treated by primary PCI. PLoS One. 2016;11(4):e0152367. doi: 10.1371/journal.pone.0152367 27074002 PMC4830576

[7] van Dijk MR, Utens EMWJ, Dulfer K, Al-Qezweny MNA, van Geuns R-J, Daemen J, et al. Depression and anxiety symptoms as predictors of mortality in PCI patients at 10 years of follow-up. Eur J Prev Cardiol. 2016;23(5):552–8. doi: 10.1177/2047487315571889 25665581

[8] Shi B, Yu B, Chen C, Liu C, Xi C, Wei C, et al. Guidelines for cognitive clinical diagnosis and treatment of coronary artery disease complicated with depression and anxiety: December 2025 update. Heart and Mind. 2025;9(6):462–71. doi: 10.4103/hm.hm-d-25-00151

[9] Sohier L, Dallaire-Habel S, Turcotte S, Foldes-Busque G. Prevalence of mood and anxiety disorders in Canadians with cardiovascular disease: A cross-sectional study. Heart and Mind. 2024;8(1):40–6. doi: 10.4103/hm.hm-d-23-00060

[10] Hadas Y, Vincek AS, Youssef E, Żak MM, Chepurko E, Sultana N, et al. Altering sphingolipid metabolism attenuates cell death and inflammatory response after myocardial infarction. Circulation. 2020;141(11):916–30. doi: 10.1161/circulationaha.119.041882 31992066 PMC7135928

[11] Jahromi GG, Rezaei N. Connecting the Dots: NLRP3 inflammasome as a key mediator in the intersection of depression and cardiovascular disease – A narrative review. Heart and Mind. 2025;9(1):48–60. doi: 10.4103/hm.hm-d-24-00076

[12] Toldo S, Abbate A. The NLRP3 inflammasome in acute myocardial infarction. Nat Rev Cardiol. 2018;15(4):203–14. doi: 10.1038/nrcardio.2017.161 29143812

[13] Pellegrini C, Martelli A, Antonioli L, Fornai M, Blandizzi C, Calderone V. NLRP3 inflammasome in cardiovascular diseases: Pathophysiological and pharmacological implications. Med Res Rev. 2021;41(4):1890–926. doi: 10.1002/med.21781 33460162

[14] Zhen Y, Zhang H. NLRP3 inflammasome and inflammatory bowel disease. Front Immunol. 2019;10:276. doi: 10.3389/fimmu.2019.00276 30873162 PMC6403142

[15] Mauro AG, Bonaventura A, Mezzaroma E, Quader M, Toldo S. NLRP3 inflammasome in acute myocardial infarction. J Cardiovasc Pharmacol. 2019;74(3):175–87. doi: 10.1097/FJC.0000000000000717 31356555

[16] Dukes ID, Vaughan Williams EM. Cardiovascular effects of bevantolol, a selective beta 1-adrenoceptor antagonist with a novel pharmacological profile. Br J Pharmacol. 1985;84(2):365–80. doi: 10.1111/j.1476-5381.1985.tb12921.x 2858236 PMC1987306

[17] Frazier SK, Moser DK, O’Brien JL, Garvin BJ, An K, Macko M. Management of anxiety after acute myocardial infarction. Heart Lung. 2002;31(6):411–20. doi: 10.1067/mhl.2002.129445 12434142

[18] Reagan-Shaw S, Nihal M, Ahmad N. Dose translation from animal to human studies revisited. FASEB J. 2008;22(3):659–61. doi: 10.1096/fj.07-9574LSF 17942826

[19] Nair AB, Jacob S. A simple practice guide for dose conversion between animals and human. J Basic Clin Pharm. 2016;7(2):27–31. doi: 10.4103/0976-0105.177703 27057123 PMC4804402

[20] Chen H, Zhou H, Yang J, Wan H, He Y. Guhong injection mitigates myocardial ischemia/reperfusion injury by activating GST P to inhibit ASK1-JNK/p38 pathway. Phytomedicine. 2023;109:154603. doi: 10.1016/j.phymed.2022.154603 36610111

[21] Granger CB, Kochar A. Understanding and targeting inflammation in acute myocardial infarction: An elusive goal. J Am Coll Cardiol. 2018; 72(2):199–201. doi: 10.1016/j.jacc.2018.05.006 29976294

[22] Crowe JM, Runions J, Ebbesen LS, Oldridge NB, Streiner DL. Anxiety and depression after acute myocardial infarction. Heart Lung. 1996;25(2):98–107. doi: 10.1016/s0147-9563(96)80111-7 8682691

[23] Liu N, Liu S, Yu N, Peng Y, Wen Y, Tang J, et al. Correlations among psychological resilience, self-efficacy, and negative emotion in acute myocardial infarction patients after percutaneous coronary intervention. Front Psychiatry. 2018;9:1. doi: 10.3389/fpsyt.2018.00001 29410632 PMC5787139

[24] Daniel M, Agewall S, Berglund F, Caidahl K, Collste O, Ekenbäck C, et al. Prevalence of anxiety and depression symptoms in patients with myocardial infarction with non-obstructive coronary arteries. Am J Med. 2018;131(9):1118–24. doi: 10.1016/j.amjmed.2018.04.040 29859805

[25] Fayyaz AI, Ding Y. Mental stress, meditation, and yoga in cardiovascular and cerebrovascular diseases. Brain Circ. 2023;9(1):1–2. doi: 10.4103/bc.bc_66_22 37151798 PMC10158665

[26] Goldberger JJ, Bonow RO, Cuffe M, Liu L, Rosenberg Y, Shah PK, et al. Effect of beta-blocker dose on survival after acute myocardial infarction. J Am Coll Cardiol. 2015;66(13):1431–41. doi: 10.1016/j.jacc.2015.07.047 26403339 PMC4583654

[27] Wong W-T, Li L-H, Rao YK, Yang S-P, Cheng S-M, Lin W-Y, et al. Repositioning of the β-blocker carvedilol as a novel autophagy inducer that inhibits the NLRP3 inflammasome. Front Immunol. 2018;9:1920. doi: 10.3389/fimmu.2018.01920 30186288 PMC6113403

[28] Lu Y, Li L, Zhao X, Huang W, Wen W. Beta blocker metoprolol protects against contractile dysfunction in rats after coronary microembolization by regulating expression of myocardial inflammatory cytokines. Life Sci. 2011;88(23–24):1009–15. doi: 10.1016/j.lfs.2011.03.012 21443890

[29] Dunn JT, Mroczek J, Patel HR, Ragozzino ME. Tandospirone, a partial 5-HT1A receptor agonist, administered systemically or into anterior cingulate attenuates repetitive behaviors in Shank3B mice. Int J Neuropsychopharmacol. 2020;23(8):533–42. doi: 10.1093/ijnp/pyaa047 32619232 PMC7689206

[30] Liu L, Yang W, Lu Y, Wang J, Zheng Y, Gu S. Clinical efficacy of tandospirone on functional dyspepsia patients with anxiety: A randomized, placebo-controlled study. Dig Dis Sci. 2023;68(2):521–8. doi: 10.1007/s10620-022-07717-z 36383269

[31] Sterpetti AV. Inflammatory cytokines and atherosclerotic plaque progression. Therapeutic implications. Curr Atheroscler Rep. 2020;22(12):75. doi: 10.1007/s11883-020-00891-3 33025148 PMC7538409

[32] Kovacs SB, Miao EA. Gasdermins: Effectors of pyroptosis. Trends in Cell Biology. 2017;27(9):673–84. doi: 10.1016/j.tcb.2017.05.005 28619472 PMC5565696

[33] Fritsch M, Günther SD, Schwarzer R, Albert M-C, Schorn F, Werthenbach JP, et al. Caspase-8 is the molecular switch for apoptosis, necroptosis and pyroptosis. Nature. 2019;575(7784):683–7. doi: 10.1038/s41586-019-1770-6 31748744

[34] Wang Y, Gao W, Shi X, Ding J, Liu W, He H, et al. Chemotherapy drugs induce pyroptosis through caspase-3 cleavage of a gasdermin. Nature. 2017;547(7661):99–103. doi: 10.1038/nature22393 28459430

[35] Shi J, Gao W, Shao F. Pyroptosis: Gasdermin-mediated programmed necrotic cell death. Trends Biochem Sci. 2017;42(4):245–54. doi: 10.1016/j.tibs.2016.10.004 27932073

[36] Zhuang Z, Gu J, Li BO, Yang L. Inhibition of gasdermin D palmitoylation by disulfiram is crucial for the treatment of myocardial infarction. Transl Res. 2023. doi: 10.1016/j.trsl.2023.09.007 37769810

[37] Weng Y, Ye B, Lin J, Lin S, Zhong L, Huang W, et al. Elevated circulating levels of gasdermin D are related to acute myocardial infarction and pyrogptosis. BMC Cardiovasc Disord. 2022;22(1):554. doi: 10.1186/s12872-022-02998-8 36544106 PMC9769051

[38] Lei Q, Yi T, Chen C. NF-κB-Gasdermin D (GSDMD) axis couples oxidative stress and NACHT, LRR and PYD Domains-Containing Protein 3 (NLRP3) inflammasome-mediated cardiomyocyte pyroptosis following myocardial infarction. Med Sci Monit. 2018;24:6044–52. doi: 10.12659/MSM.908529 30161099 PMC6128186

[39] Johnson J. Effect of emotions on learning, memory, and disorders associated with the changes in expression levels: A narrative review. Brain Circ. 2024;10(2):134–44. doi: 10.4103/bc.bc_86_23 39036298 PMC11259327

[40] Brunoni AR, Supasitthumrong T, Teixeira AL, Vieira EL, Gattaz WF, Benseñor IM, et al. Differences in the immune-inflammatory profiles of unipolar and bipolar depression. J Affect Disord. 2020;262:8–15. doi: 10.1016/j.jad.2019.10.037 31693974

[41] Hou R, Garner M, Holmes C, Osmond C, Teeling J, Lau L, et al. Peripheral inflammatory cytokines and immune balance in Generalised Anxiety Disorder: Case-controlled study. Brain Behav Immun. 2017;62:212–8. doi: 10.1016/j.bbi.2017.01.021 28161475 PMC5373436

[42] Lei W-X, Zhang L, Chen J-L, Zheng G-H, Guo L-N, Jiang T, et al. The role and mechanism of miR-425-3p regulating neuronal pyroptosis -mediated inorganic arsenic-induced generalized anxiety disorder. Ecotoxicol Environ Saf. 2024;269:115781. doi: 10.1016/j.ecoenv.2023.115781 38056122

[43] Miao EA, Rajan JV, Aderem A. Caspase-1-induced pyroptotic cell death. Immunol Rev. 2011;243(1):206–14. doi: 10.1111/j.1600-065X.2011.01044.x 21884178 PMC3609431

