# Supplementary material for: Protection of beta-blocker and 5-hydroxytryptamine 1A receptor agonist on myocardial ischemia injury in rats by regulating the NLRP3/Caspase-1/GSDMD pathway
Source: PLoS One. 2026 Oct 14;21(10):e0360695. doi: 10.1371/journal.pone.0360695 (PMC13667816; doi:10.1371/journal.pone.0360695)
Supplement: Supplementary file 1 [file pone.0360695.s007.pdf]

Original Western blot for Figure 5C

IL-18:

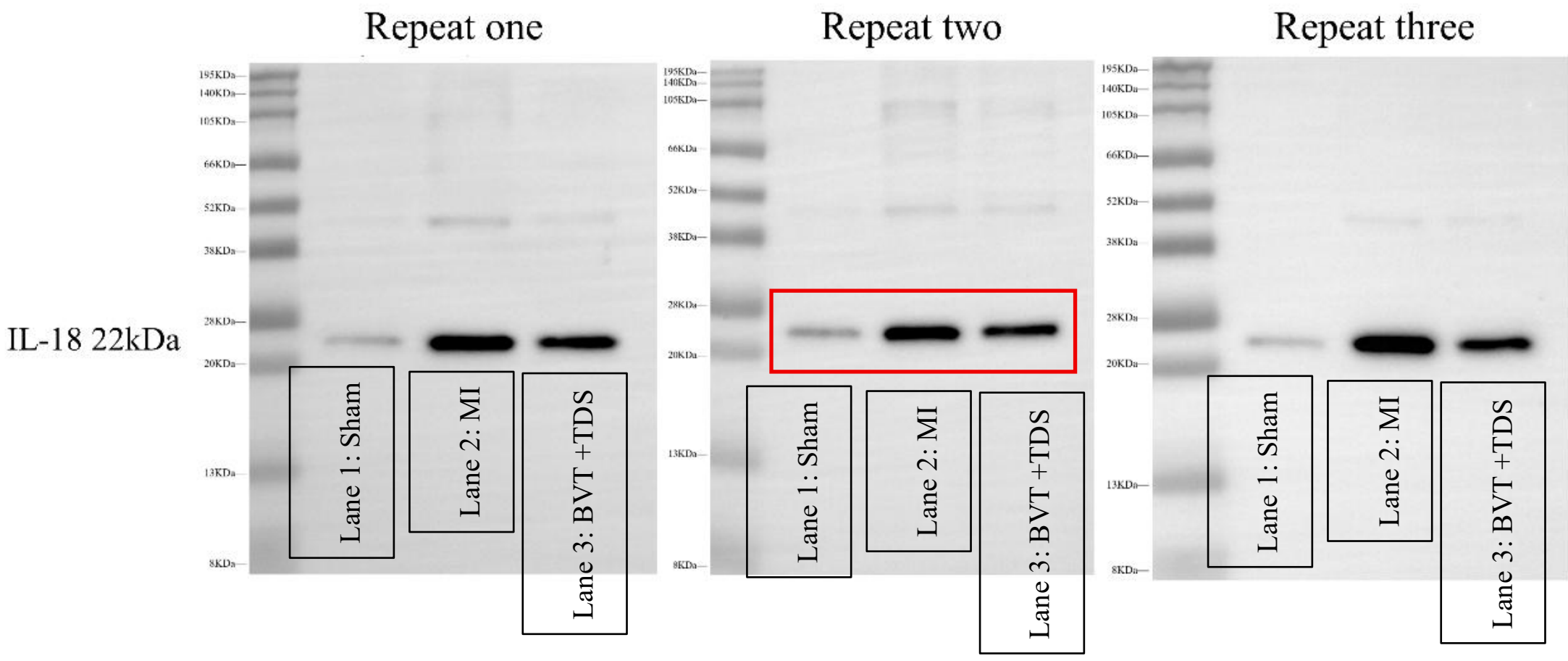

Primary: anti-IL-18 (Cat., AF5103, 1: 1000, Affinity)

Secondary: HRP-Goat anti-Rabbit (Cat., RGAR001, 1: 5000, Proteintech)

Exposure: Chemiluminescence imaging system (SCG-W2000, ServiceBio)

IL-1β:

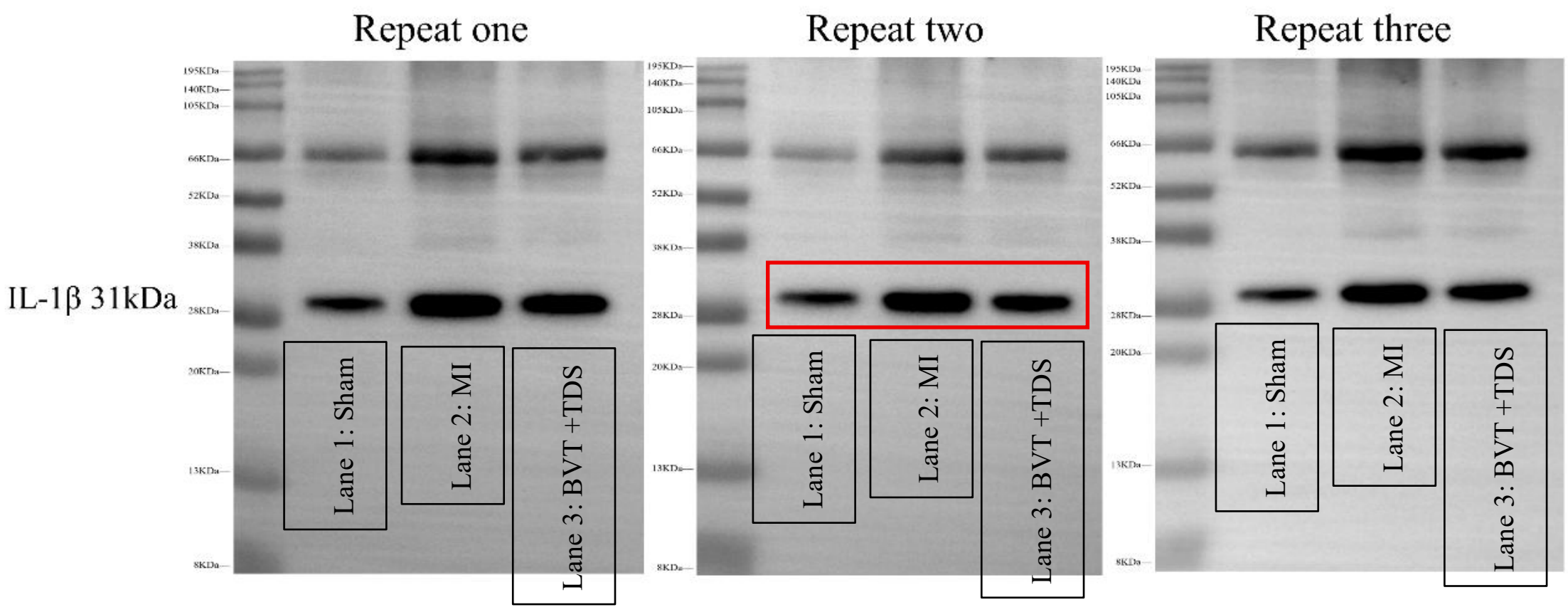

Primary: anti-IL-1β (Cat., DF6252, 1: 1000, Affinity)

Secondary: HRP-Goat anti-Rabbit (Cat., RGAR001, 1: 5000, Proteintech)

Exposure: Chemiluminescence imaging system (SCG-W2000, ServiceBio)

GAPDH:

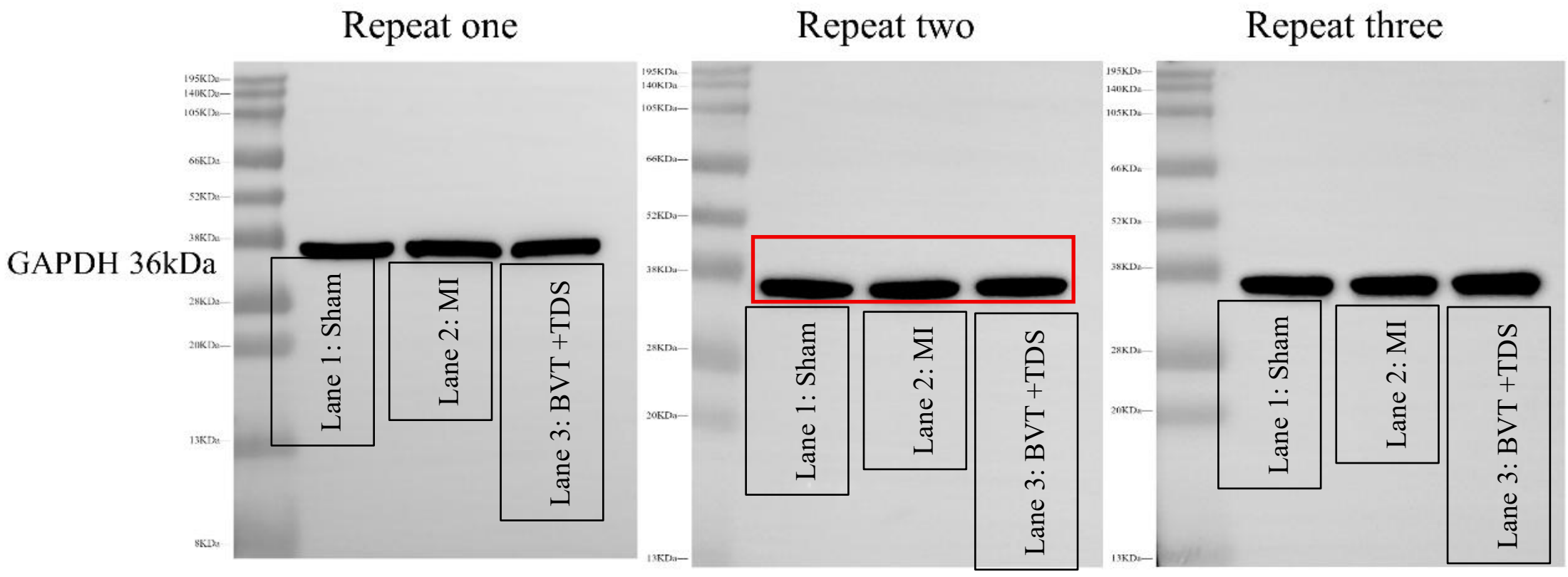

Primary: anti-GAPDH (Cat., 60004-1-lg, 1: 3000, Proteintech)

Secondary: HRP-Goat anti-Rabbit (Cat., RGAR001, 1: 5000, Proteintech)

Exposure: Chemiluminescence imaging system (SCG-W2000, ServiceBio)

Original Western blot for Figure 5E

pro Caspase-1

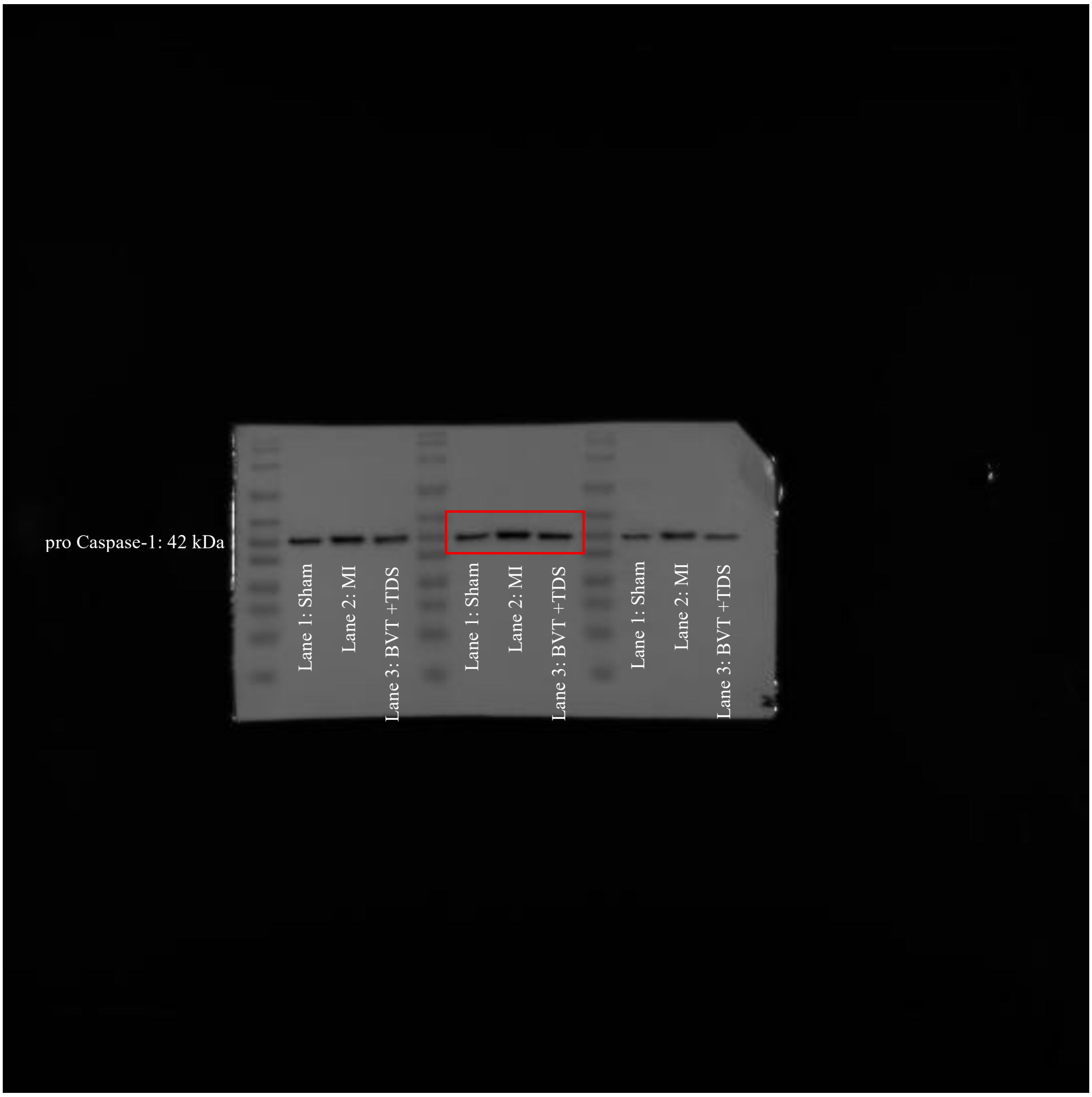

Primary: anti-pro Caspase-1 (Cat., ab179515, 1: 1000, Abcam)

Secondary: HRP-Goat anti-Rabbit (Cat., RGAR001, 1: 5000, Proteintech)

Exposure: Chemiluminescence imaging system (SCG-W2000, ServiceBio)

GAPDH:

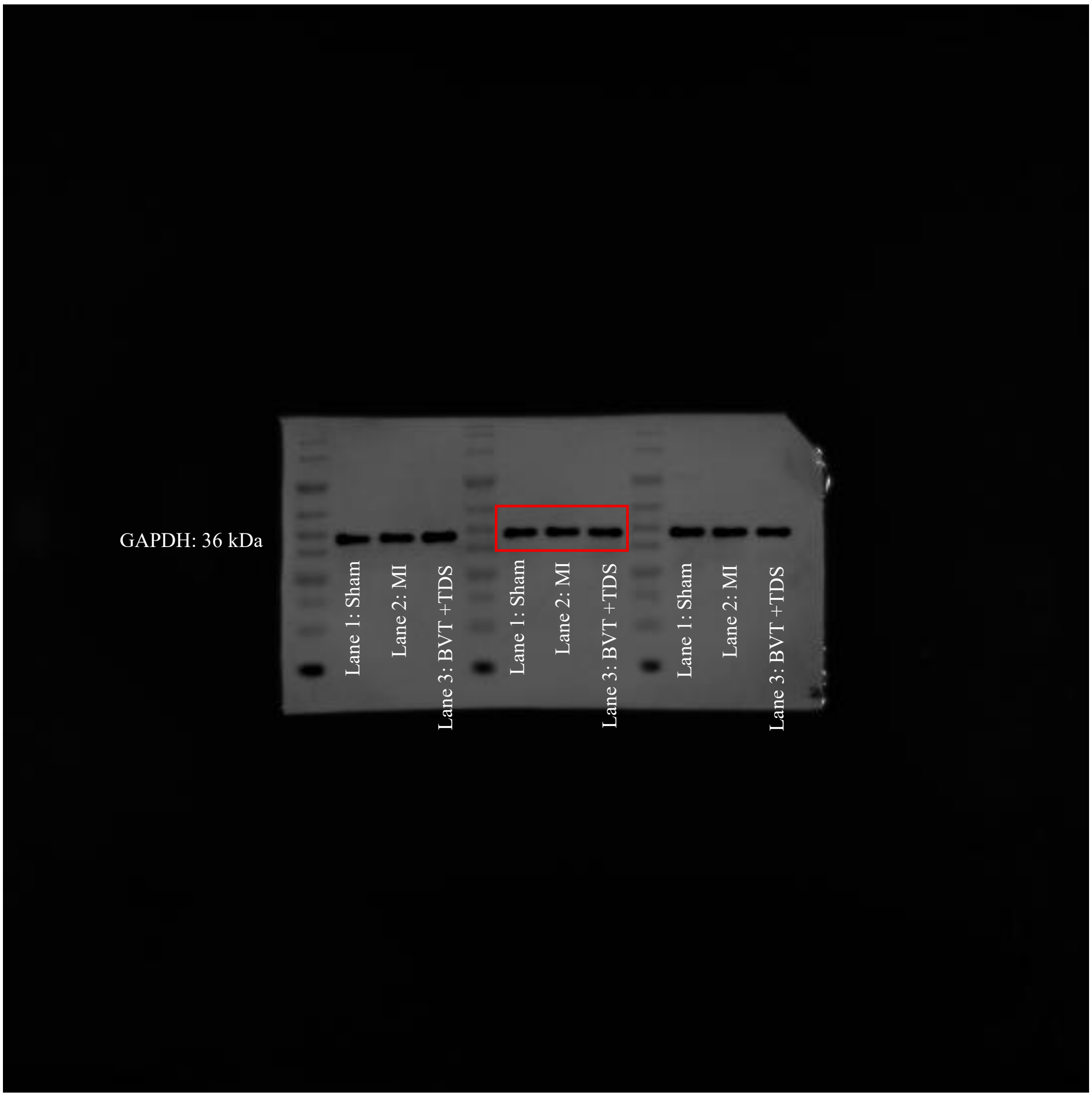

Primary: anti-GAPDH (Cat., 60004-1-lg, 1: 3000, Proteintech)

Secondary: HRP-Goat anti-Rabbit (Cat., RGAR001, 1: 5000, Proteintech)

Exposure: Chemiluminescence imaging system (SCG-W2000, ServiceBio)

GSDMD-FL

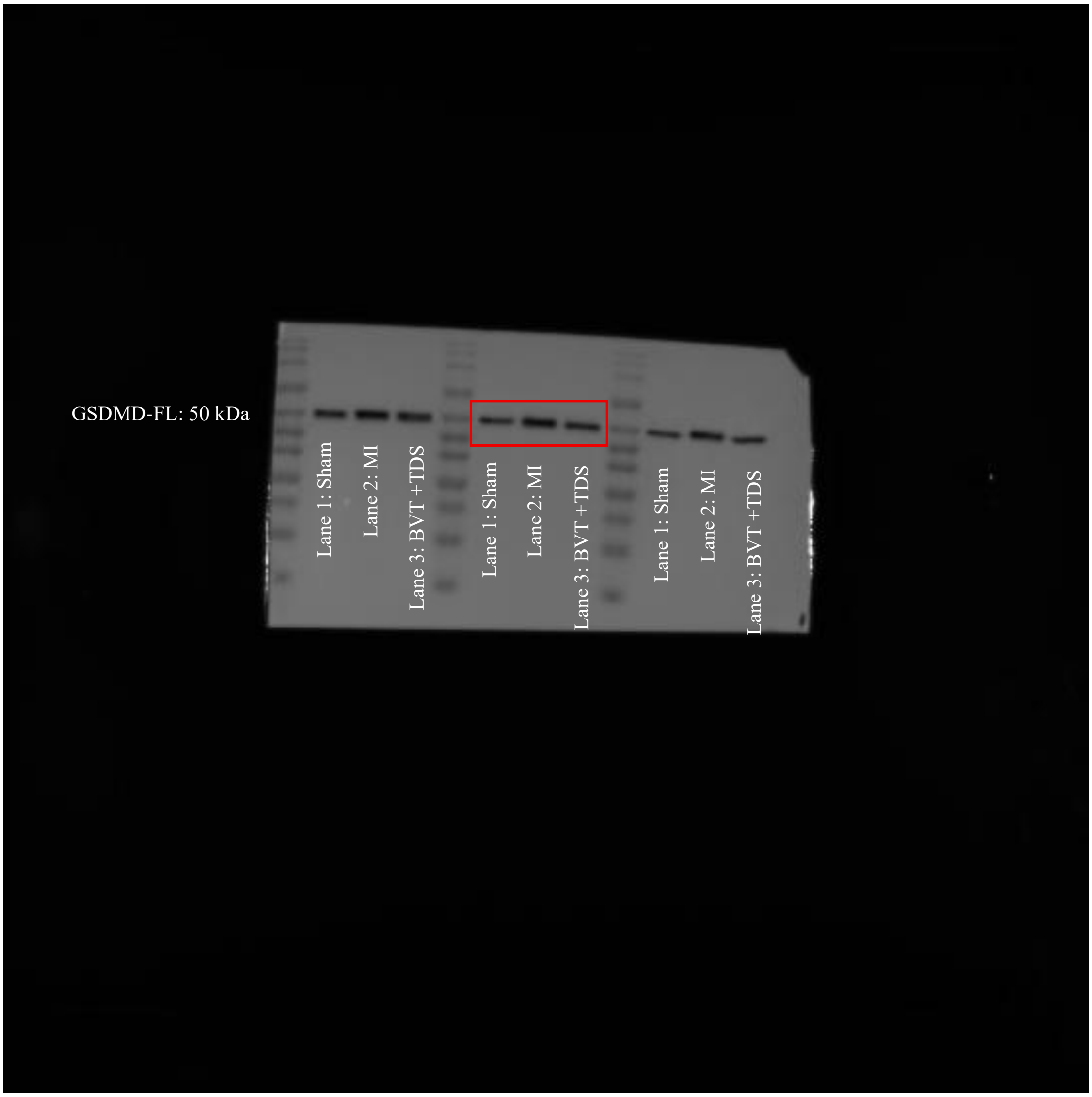

Primary: anti-GSDMD-FL (Cat., 20770-1-AP, 1: 2000, Proteintech)

Secondary: HRP-Goat anti-Rabbit (Cat., RGAR001, 1: 5000, Proteintech)

Exposure: Chemiluminescence imaging system (SCG-W2000, ServiceBio)

GAPDH

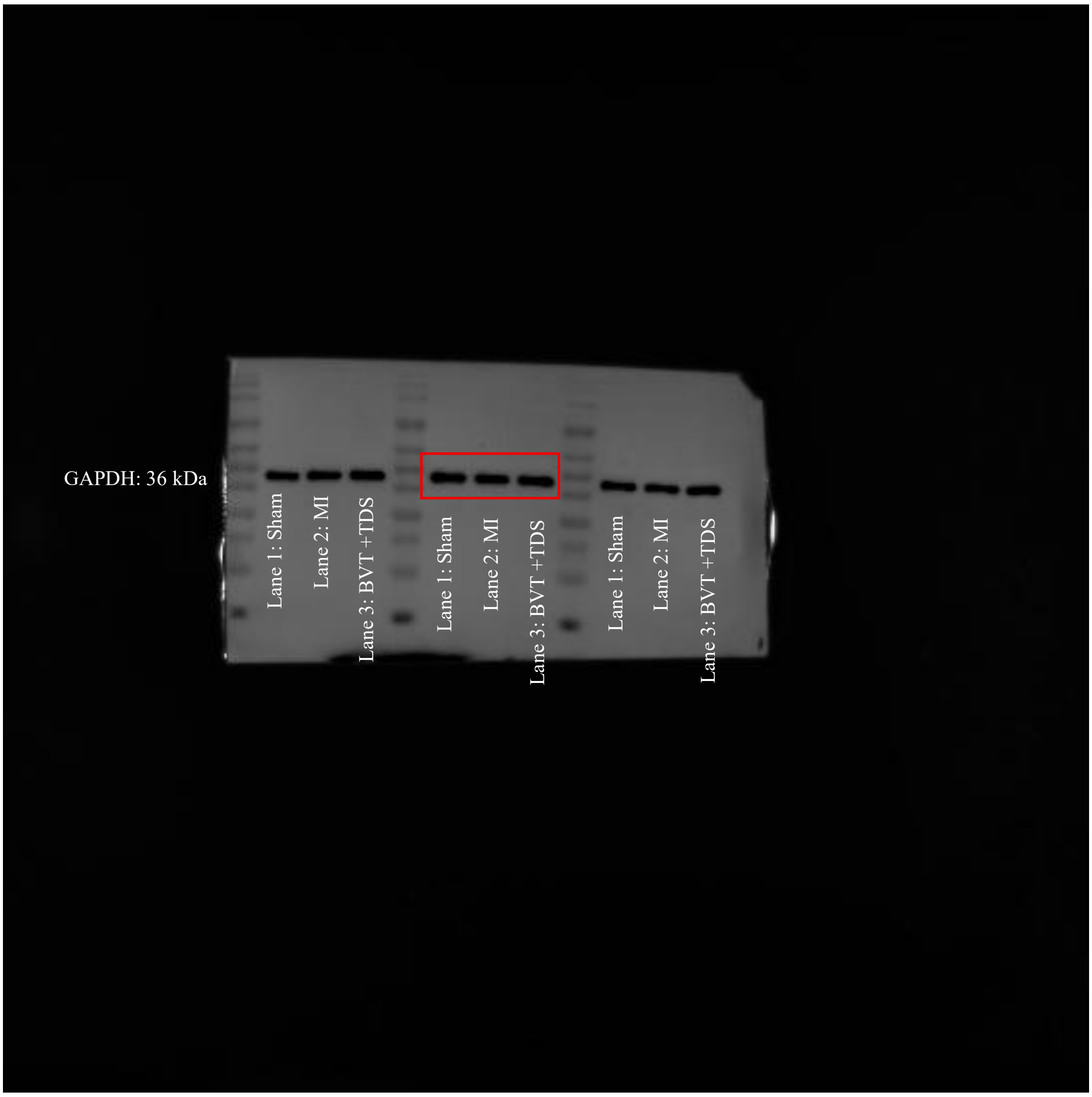

Primary: anti-GAPDH (Cat., 60004-1-lg, 1: 3000, Proteintech)

Secondary: HRP-Goat anti-Rabbit (Cat., RGAR001, 1: 5000, Proteintech)

Exposure: Chemiluminescence imaging system (SCG-W2000, ServiceBio)

Original Western blot for Figure 5G

NLRP3:

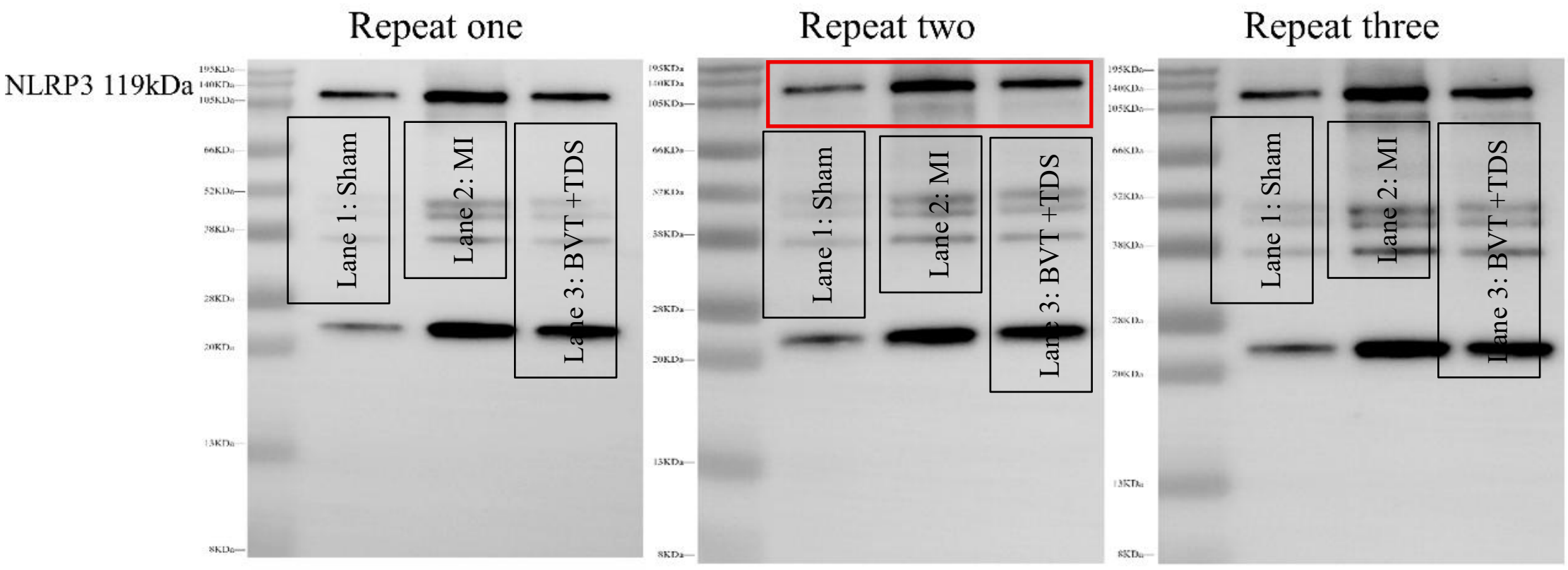

Primary: anti-NLRP3 (Cat., DF15549, 1: 1000, Affinity)

Secondary: HRP-Goat anti-Rabbit (Cat., RGAR001, 1: 5000, Proteintech)

Exposure: Chemiluminescence imaging system (SCG-W2000, ServiceBio)

cleaved Caspase-1:

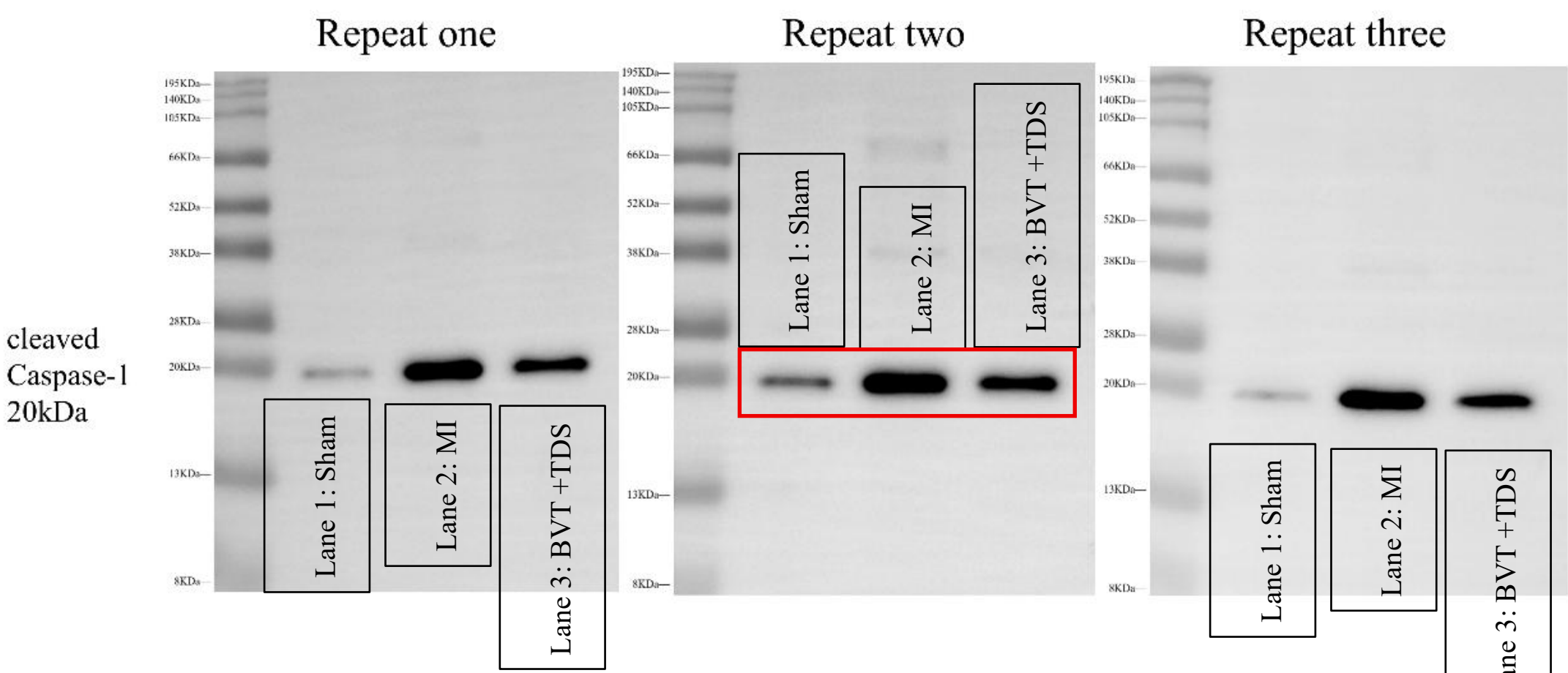

Primary: anti-cleaved Caspase-1 (Cat., AF4005, 1: 1000, Affinity)

Secondary: HRP-Goat anti-Rabbit (Cat., RGAR001, 1: 5000, Proteintech)

Exposure: Chemiluminescence imaging system (SCG-W2000, ServiceBio)

GSDMD-N:

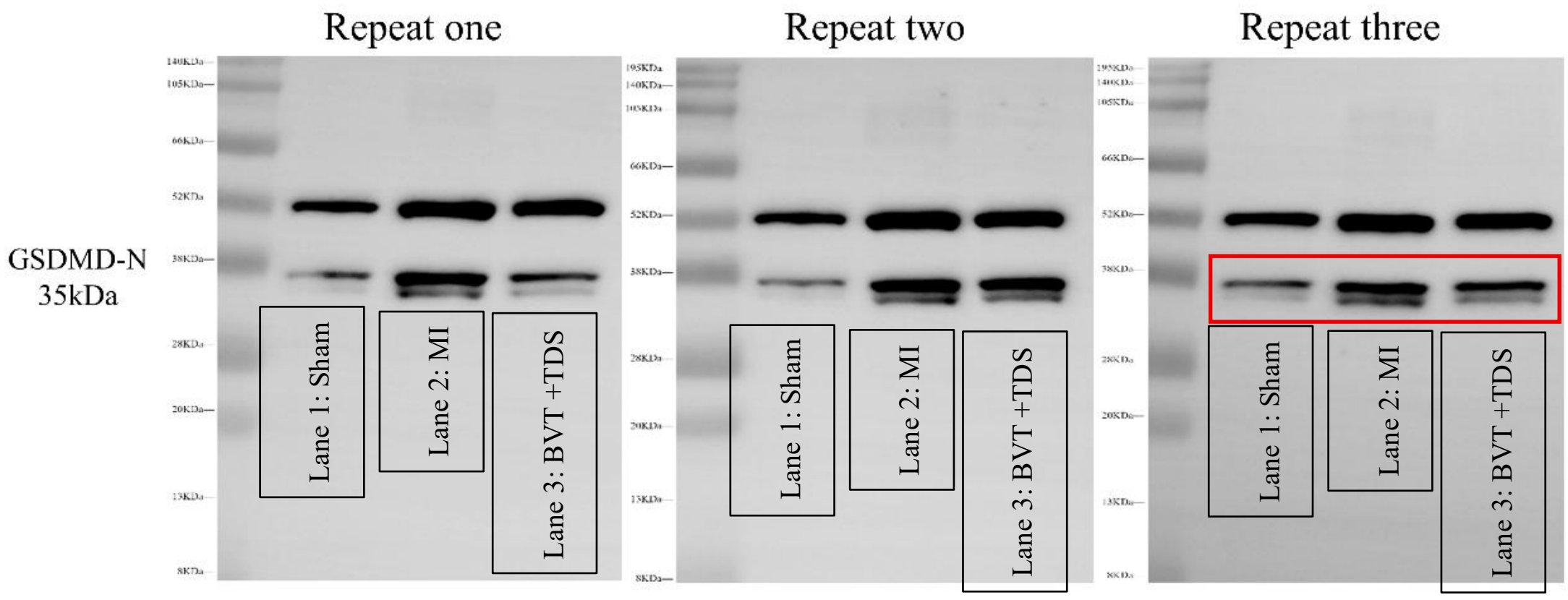

Primary: anti-GSDMD-N (Cat., DF13758, 1: 1000, Affinity)

Secondary: HRP-Goat anti-Rabbit (Cat., RGAR001, 1: 5000, Proteintech)

Exposure: Chemiluminescence imaging system (SCG-W2000, ServiceBio)

GAPDH:

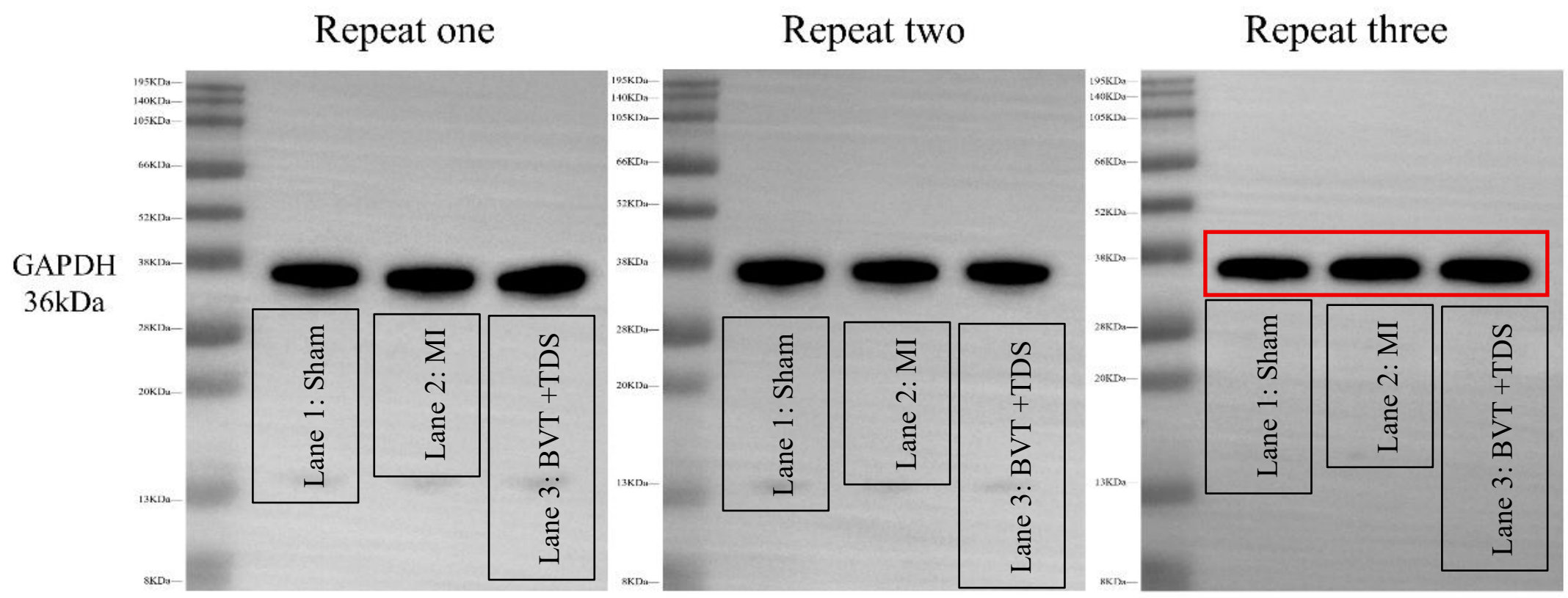

Primary: anti-GAPDH (Cat., 60004-1-lg, 1: 3000, Proteintech)

Secondary: HRP-Goat anti-Rabbit (Cat., RGAR001, 1: 5000, Proteintech)

Exposure: Chemiluminescence imaging system (SCG-W2000, ServiceBio)
